# Implications of alternative routes to APC/C inhibition by the mitotic checkpoint complex

**DOI:** 10.1371/journal.pcbi.1006449

**Published:** 2018-09-10

**Authors:** Fridolin Gross, Paolo Bonaiuti, Silke Hauf, Andrea Ciliberto

**Affiliations:** 1 Istituto Firc di Oncologia Molecolare, IFOM, Milano, Italy; 2 Department of Biological Sciences, Virginia Tech, Blacksburg, VA, United States of America; 3 Biocomplexity Institute, Virginia Tech, Blacksburg, VA, United States of America; 4 Center for Soft Matter and Biological Physics, Virginia Tech, Blacksburg, VA, United States of America; 5 Istituto di Genetica Molecolare, Consiglio Nazionale delle Ricerche (IGM-CNR), Pavia, Italy; Ecole Normale Supérieure, FRANCE

## Abstract

The mitotic checkpoint (also called spindle assembly checkpoint) is a signaling pathway that ensures faithful chromosome segregation. Mitotic checkpoint proteins inhibit the anaphase-promoting complex (APC/C) and its activator Cdc20 to prevent precocious anaphase. Checkpoint signaling leads to a complex of APC/C, Cdc20, and checkpoint proteins, in which the APC/C is inactive. In principle, this final product of the mitotic checkpoint can be obtained via different pathways, whose relevance still needs to be fully ascertained experimentally. Here, we use mathematical models to compare the implications on checkpoint response of the possible pathways leading to APC/C inhibition. We identify a previously unrecognized funneling effect for Cdc20, which favors Cdc20 incorporation into the inhibitory complex and therefore promotes checkpoint activity. Furthermore, we find that the presence or absence of one specific assembly reaction determines whether the checkpoint remains functional at elevated levels of Cdc20, which can occur in cancer cells. Our results reveal the inhibitory logics behind checkpoint activity, predict checkpoint efficiency in perturbed situations, and could inform molecular strategies to treat malignancies that exhibit Cdc20 overexpression.

## Author summary

Cell division is a fundamental event in the life of cells. It requires that a mother cell gives rise to two daughters which carry the same genetic material of their mother. Thus, during each cell cycle the genetic material needs to be replicated, compacted into chromosomes and redistributed to the two daughter cells. Any mistake in chromosome segregation would attribute the wrong number of chromosomes to the progeny. Hence, the process of chromosome segregation is closely watched by a surveillance mechanism known as the mitotic checkpoint. The molecular players of the checkpoint pathway are well known: we know both the input (ie, the species to be inhibited and their inhibitors), and the output (ie, the inhibited species). However, we do not exactly know the path that leads from the former to the latter. In this manuscript, we use a mathematical approach to explore the properties of plausible mitotic checkpoint networks. We find that seemingly similar circuits show very different behaviors for high levels of the protein targeted by the mitotic checkpoint, Cdc20. Interestingly, this protein is often overexpressed in cancer cells. For physiological levels of Cdc20, instead, all the models we have analyzed are capable to mount an efficient response. We find that this is due to a series of consecutive protein-protein binding reactions that funnel Cdc20 towards its inhibited state. We call this the funneling effect. Our analysis helps understanding the inhibitory logics underlying the checkpoint, and proposes new concepts that could be applied to other inhibitory pathways.

## Introduction

Faithful chromosome segregation requires that each sister chromatid moves towards a different daughter cell. This is guaranteed by the mitotic checkpoint (also known as spindle assembly checkpoint or SAC). As long as chromosomes are improperly attached to the mitotic spindle, the mitotic checkpoint inhibits the anaphase-promoting complex (APC/C) and its essential coactivator Cdc20, thereby preventing anaphase. Only when all attachments are correct, the checkpoint is lifted: APC/C^Cdc20^ becomes active, chromosome segregation takes place and now has a high chance of being executed correctly [1].

**Fig 1:**
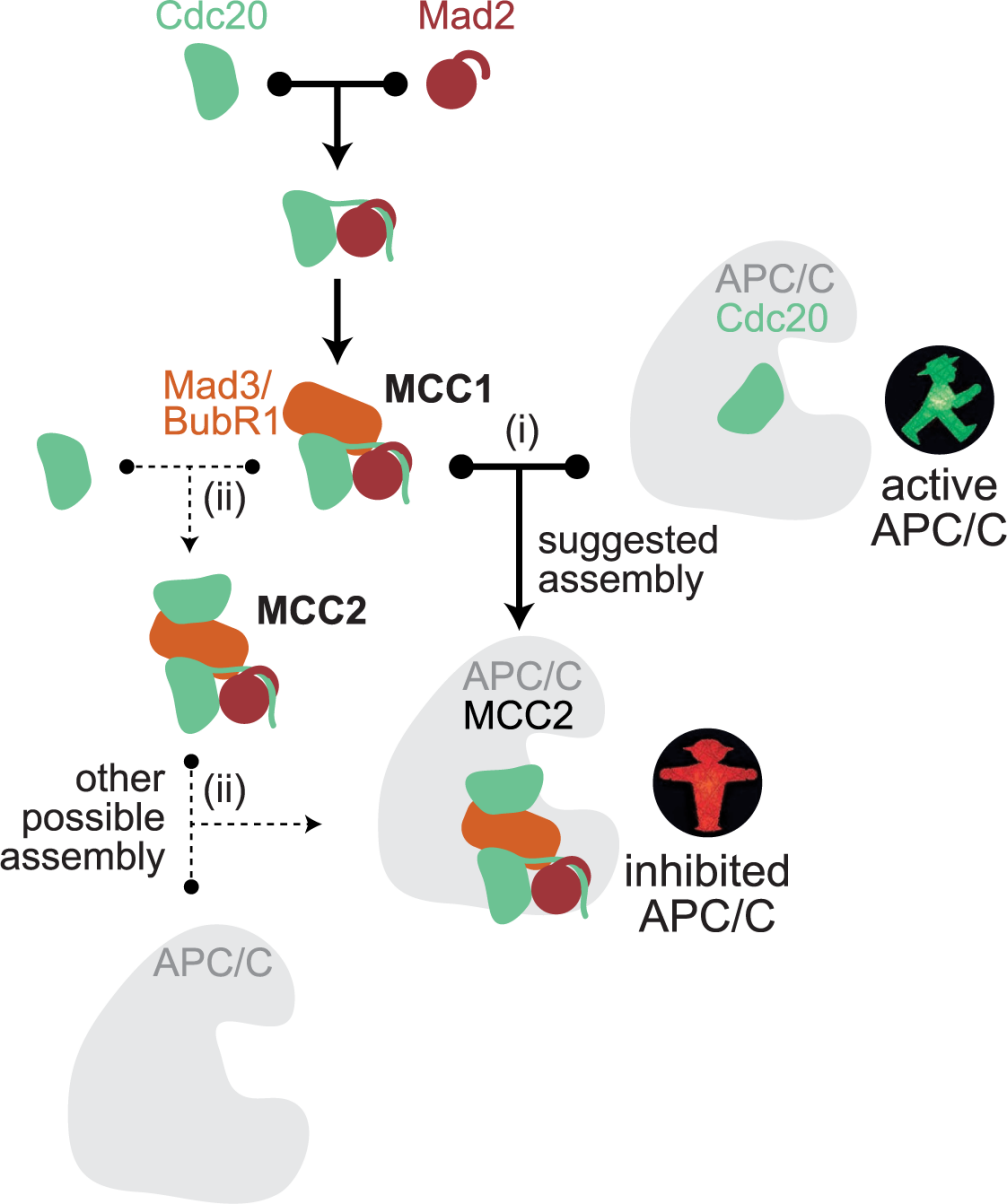
Wiring diagram of possible reactions leading to APC/C^MCC2^ (inhibited APC/C) formation. APC/C^Cdc20^ is the active form of the APC/C that initiates anaphase. Reaction (i) indicates APC/C^MCC2^ formation by MCC1 binding to APC/C^Cdc20^. Reactions marked with (ii) indicate APC/C^MCC2^ formation by MCC2 binding to APC/C.

The mitotic checkpoint network has been investigated for decades, and likely all of the relevant players have been identified [1, 2]. Models have been developed to analyze important systems level features of this surveillance mechanisms [3–9]. The network consists of a cascade of association/dissociation reactions, which culminates in the formation of the inhibited form of the APC/C. The ultimate effector of APC/C inhibition is the mitotic checkpoint complex (MCC). The network of reactions leading to MCC assembly is complex and involves kinetochore proteins, the mitotic checkpoint kinase Mps1/Mph1/TTK, as well as the checkpoint proteins Bub1, Bub3, BubR1/Mad3, Mad1 and Mad2 [1, 2, 10]. The rate-limiting step for MCC formation is the binding of Mad2 to Cdc20, which initiates MCC assembly. This reaction is catalyzed by a tight complex between the Mad1 and Mad2 proteins [11–14]. Mad1-bound Mad2 dimerizes with free Mad2, which renders the latter competent for Cdc20 binding [15–17]. A complex of Mad3/BubR1 and Bub3 then binds the Mad2-Cdc20 complex to form the MCC [13, 18–20]. The MCC in turn binds the APC/C, a ubiquitin ligase, and prevents it from targeting its anaphase substrates–mitotic cyclins and securin–for degradation. The APC/C-MCC complex contains two molecules of Cdc20, not only one as had been supposed for decades [21, 22]. It is unclear whether MCC that is not bound to the APC/C can also contain two molecules of Cdc20. We refer to the two possible MCC complexes with one or two Cdc20 molecules as MCC1 and MCC2, respectively (Fig 1). In the APC/C-MCC complex, the two Cdc20 molecules are bound to two different KEN box motifs in the checkpoint protein Mad3/BubR1 [21–26]. Crucially, binding of both Cdc20 molecules is needed for APC/C inhibition [18, 25–31].

These results have been solidly confirmed by multiple studies in several organisms, suggesting that we have reached a consensus picture of the main molecular actors in the mitotic checkpoint pathway. Yet, the actual series of events leading to formation of APC/C^MCC2^ is not unambiguously defined, and it is far from trivial to experimentally decide between alternative intermediates [32]. Here, we build computational models for the possible pathways that lead from Mad2-Cdc20 binding to APC/C^MCC2^ formation (Fig 1) and ask about the inherent characteristics of the resulting networks. This leads us to identify core features of checkpoint signaling that have not been recognized previously: (i) a funneling effect for Cdc20 that gives it a preference for incorporation into inhibitory complexes, and (ii) identification of free MCC2 formation as a key reaction that determines whether or not the mitotic checkpoint is functional at high Cdc20 concentrations. Knowledge of these features will make it easier to predict and purposefully modulate mitotic checkpoint behavior.

## Results

### APC/C^MCC2^ may be formed through different intermediates

The final product of mitotic checkpoint signaling is the MCC-inhibited form of the APC/C, which carries two molecules of Cdc20 (APC/C^MCC2^) [22, 23, 26]. APC/C^MCC2^ formation may follow two paths, which are not mutually exclusive: (i) as has been suggested [22], APC/C^MCC2^ is created by binding of MCC1 to APC/C^Cdc20^; or (ii) the free MCC2 complex binds to the APC/C (Fig 1). Theoretically, there is a third possibility that binding of MCC1 to APC/C gives rise to APC/C^MCC1^, which then picks up a second molecule of Cdc20 to become APC/C^MCC2^. However, MCC1 binds the APC/C poorly when binding sites for the second Cdc20 molecule are mutated [25, 28, 33]. This suggests that APC/C^MCC1^ is unstable, and unlikely to be a precursor for APC/C^MCC2^. We therefore do not include this last possibility in our analysis.

**Fig 2:**
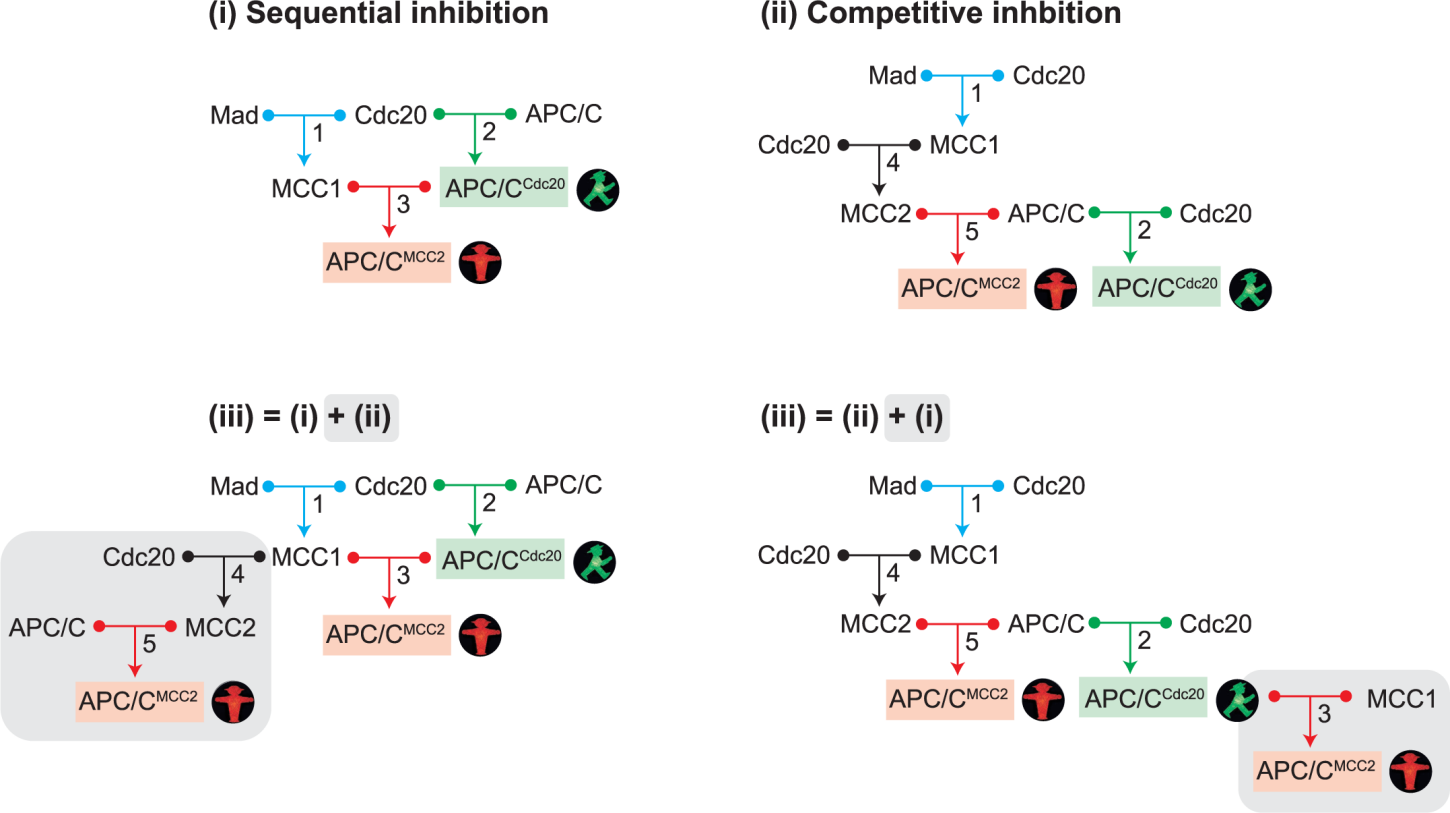
Different networks for APC/C^MCC2^ formation. Free Mad2 and Mad3 were combined in the variable ‘Mad’. (i) Sequential inhibition network, which is characterized by MCC1 binding to APC/C^Cdc20^. (ii) Competitive inhibition network, which is characterized by competition between MCC2 and Cdc20 for APC/C binding. (iii) Different representations of the same mixed network, containing the reactions of both (i) and (ii). Network (iii) can be generated either by adding reaction 4 and 5 (MCC2 formation and MCC2 binding to APC/C) to the sequential inhibition network, or by adding reaction 3 (MCC1 binding to APC/C^Cdc20^) to the competitive inhibition network. Note that in networks (ii) and (iii), some molecular species are listed more than once at different positions in order to simplify the depiction of the overall network structure.

From the remaining two possibilities of forming APC/C^MCC2^, we can assemble three networks (Fig 2). The two reactions common to all of them are the formation of MCC1 (reaction 1) and of APC/C^Cdc20^ (reaction 2). In network (i), APC/C^MCC2^ is directly formed from MCC1 and APC/C^Cdc20^ (reaction 3), whereas in network (ii) MCC1 first binds a second Cdc20 to form MCC2 (reaction 4) which in turn binds to free APC/C (reaction 5). Network (iii) includes the reactions of both (i) and (ii). One can arrive at network (iii) either by adding reactions 4 and 5 to network (i), or by adding reaction 3 to network (ii). In order to make the comparison between the networks easier, we depict both versions in Fig 2.

Experimental evidence clearly supports possibility (i) [22], but the formation of free MCC2, i.e. not bound to the APC/C, has not been ruled out. Free MCC2 has been observed after deleting the APC/C subunit Apc15 in fission yeast [24, 25]. This leaves network (i) and the combined network (iii) as the most likely possibilities. Yet, to obtain a systematic understanding of the system, we also analyze network (ii).

In network (i) (Fig 2), inhibitory binding follows the formation of the active APC/C^Cdc20^ complex. We term this ‘sequential inhibition’. In network (ii) (Fig 2), the APC/C can either associate with MCC2 to form inhibited APC/C, or with Cdc20 to form active APC/C, but active APC/C^Cdc20^ never gets converted into inactive APC/C^MCC2^. Hence, MCC2 and Cdc20 compete for the APC/C. We term this ‘competitive inhibition’. Finally, when (i) and (ii) are combined in network (iii) (Fig 2), there is still the element of sequential inhibition, but Cdc20 and MCC2 now also compete for the APC/C. It is not obvious whether all these networks can be expected to mount a mitotic checkpoint-mediated arrest, and how they will respond to perturbations.

### Checkpoint functionality is defined as the ability to suppress APC/C^Cdc20^ at steady state

We formulate mathematical models for the networks (i) to (iii) based on the law of mass action (S1 Text). We do not explicitly model Bub3, because it is in a stable complex with Mad3/BubR1 [34, 35] through which it enters the MCC [36, 37]. Cellular Mad2 is either in a stable complex with Mad1 or is in a free form that can interact with Cc20 [38]. For our model, we combine free Mad2 and Mad3/BubR1 into one species (Mad). Hence, in the model, MCC1 is created by one binding reaction (Mad+Cdc20; Fig 2) rather than two (Mad2+Cdc20 -> Mad2:Cdc20; Mad2:Cdc20+Mad3/BubR1 -> MCC1; Fig 1). This choice simplifies our calculations.

Cdc20 is known to be actively produced and degraded during a checkpoint arrest, but knowledge on the details of this synthesis and degradation is incomplete [2]. We opted to focus on the steady state behavior of the mitotic checkpoint network where Cdc20 synthesis and degradation are balanced. Different levels of Cdc20 thus correspond to situations with different rates of synthesis and degradation. The choice of performing a steady state analysis and neglecting transient dynamics of the system is based on the numerous uncertainties that still impair our understanding of the processes of Cdc20 synthesis and degradation. By choosing total Cdc20 as a bifurcation parameter, we circumvent the need for a molecular description of the mechanisms that control Cdc20 synthesis and degradation. In principle, Cdc20 degradation influences the steady state behavior of the species from which it is degraded. However, Cdc20 degradation [39–41] is typically slower than dissociation reactions of the SAC network [14, 42]. As such, in first approximation the steady state behavior can be analyzed without degradation. This steady state analysis comes at a price: it neglects transient behaviors before the system reaches the steady state. Yet, despite this limitation, the steady state is relevant since it corresponds to the situation of checkpoint arrest.

We analyze the steady state behavior for different total Cdc20 concentrations. Since Cdc20 acts both as a direct activator of APC/C, but is also involved in the inhibitory complex, it is not intuitively obvious how the system will react to changes in its concentration. Furthermore, Cdc20 levels are highly regulated, unlike most other checkpoint components, and Cdc20 is known to be overexpressed in several cancers [43–47], which calls for an investigation on the effect of high levels of Cdc20 on mitotic checkpoint activity.

We evaluate the status of the mitotic checkpoint by monitoring the steady state levels of active and inhibited forms of the APC/C: APC/C^Cdc20^ and APC/C^MCC2^, respectively. Preventing anaphase requires a strong knock-down of Cdc20 [48, 49]. We thus interpret a low concentration of APC/C^Cdc20^ as a state of mitotic checkpoint proficiency.

In summary, we will perform a steady state analysis, which allows us to draw general conclusions about checkpoint-arrested conditions that are independent of uncertainties about the molecular details of Cdc20 synthesis and degradation.

### Analytical results for limiting Cdc20 levels reveal a funneling effect

A constant concern in mathematical models is the choice of parameter values. We therefore first describe analytical results which require approximations but do not depend on a specific set of parameters (the complete analysis can be found in S1 Text). Later, we validate these approximations by using numerical simulations, for which we use parameter ranges based on experimental data.

**Fig 3:**
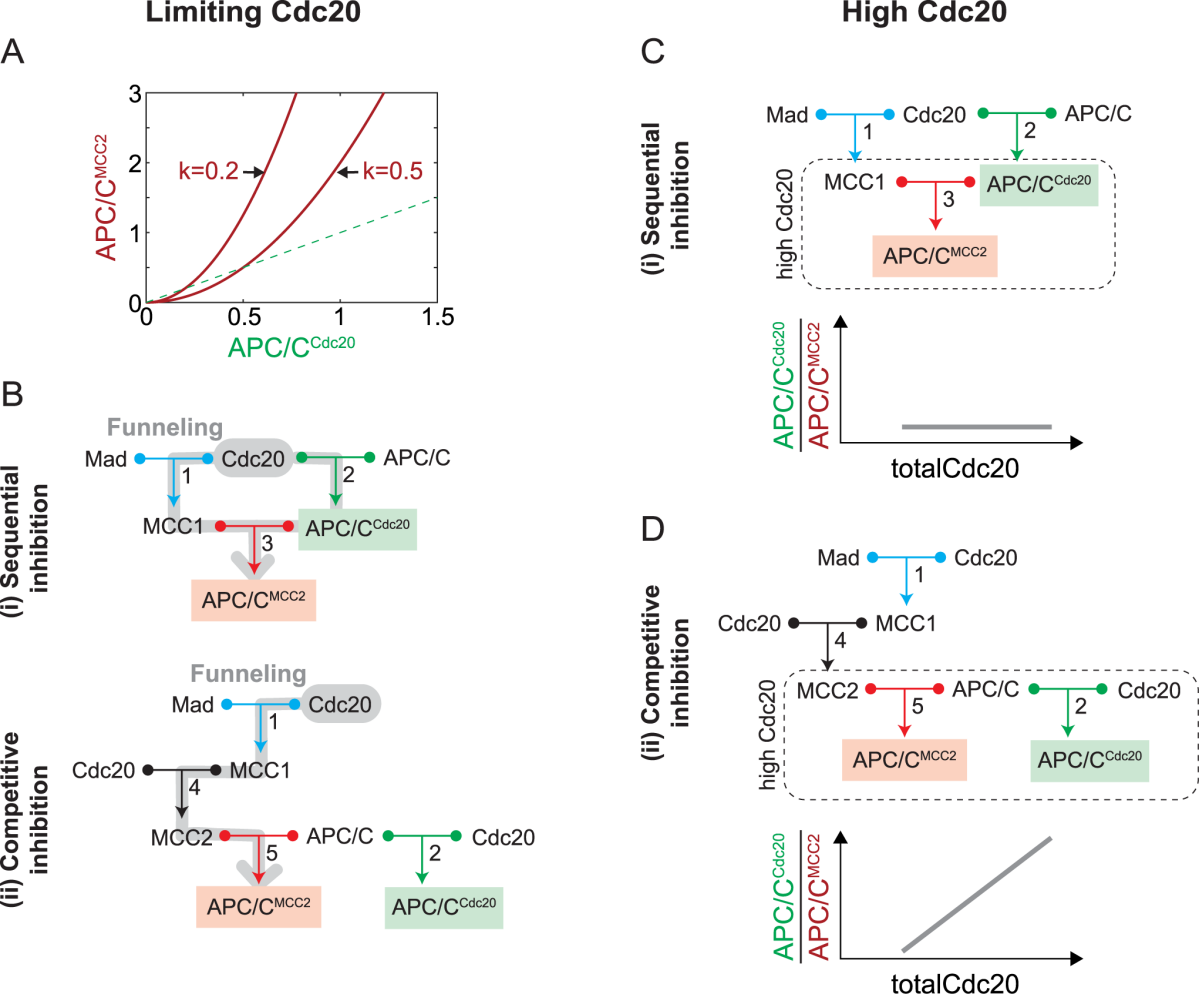
Approximations for limiting Cdc20 (Cdc20_total_<Mad_total_, APC/C_total_) or high Cdc20 (Cdc20_total_>Mad_total_, APC/C_total_) concentrations. **(A)** At limiting Cdc20 concentrations, APC/C^MCC2^ exceeds APC/C^Cdc20^ for APC/C^Cdc20^>k. The values for k are illustrative and not meant to be physiologically relevant. **(B)** Schematic representation of the funneling effect (grey path), which results in preferred APC/C^MCC2^ formation over APC/C^Cdc20^. In both sequential and competitive inhibition three binding reactions are required for APC/C^MCC2^ formation (grey), whereas only one single binding reaction (reaction 2) is required for APC/C^Cdc20^ formation. **(C)** At high Cdc20 concentrations, the sequential inhibition model reduces to reaction 3 (boxed region). In this regime, the ratio of APC/C^Cdc20^ to APC/C^MCC2^ does not depend on Cdc20 concentration (bottom graph, S1 Text, eq. 29). **(D)** At high Cdc20 concentrations, the competitive inhibition model reduces to reactions 2 and 5 (boxed region) and the ratio of APC/C^Cdc20^ to APC/C^MCC2^ increases linearly with Cdc20 concentration (bottom graph, S1 Text, eq. 64).

We obtained analytical results for two extreme conditions: Cdc20 being limiting compared to Mad and APC/C (Cdc20_total_<Mad_total_, APC/C_total_), or Cdc20 being in excess (Cdc20_total_>Mad_total_, APC/C_total_). Limiting Cdc20 implies that most Mad and APC/C remain in their free form. Thus, we can approximate free APC/C with total APC/C and free Mad with total Mad. In the sequential inhibition model (i) this approximation implies that the inhibited species scales as the square of the active species (S1 Text, eq 22):

$${APC/C}^{MCC2}=\frac{{(APC/C}^{Cdc20}{)}^{2}}{k}\;\mathrm{with}\;k=\frac{APC/C_{total}∙K_{D}^{1}∙K_{D}^{3}}{Mad_{total}∙K_{D}^{2}},$$

where $K_{D}^{1},\;K_{D}^{2}$ and $K_{D}^{3}$ are the dissociation constants of reactions 1, 2, and 3, respectively. While both APC/C^MCC2^ and APC/C^Cdc20^ increase with the total amount of Cdc20, the equation shows that APC/C^MCC2^ dominates over APC/C^Cdc20^ when APC/C^Cdc20^ > *k* (Fig 3A). This means that there is a range where Cdc20 is preferentially incorporated into the inactive complex. Both the lower limit of this range and the ratio of active to inactive APC/C depends on the value of *k*. The smaller this value, the larger the range and the stronger the dominance of inactive APC/C (Fig 3A). The value of k will be small if binding is strong (i.e. small *K*_*D*_s).

The reason for the preferential incorporation of Cdc20 into APC/C^MCC2^ is a ‘funneling effect’, which results from three binding reactions (reactions 1, 2, 3) being involved in creating inactive APC/C^MCC2^, whereas only one reaction (reaction 2) is required to make active APC/C^Cdc20^ (Fig 3B). Since the effective binding strength of the combined reactions adds up, Cdc20 is funneled into the inhibitory pathway. Interestingly, the same funneling is found in the competitive inhibition model (S1 Text, eq 44), even though MCC2 and Cdc20 equally compete for the APC/C (Fig 2). This is because, regardless of the different topologies, in both competitive and sequential inhibition models APC/C^MCC2^ formation requires three reactions, whereas APC/C^Cdc20^ formation requires a single reaction (Fig 3B).

In summary, in the Cdc20-limiting regime APC/C^MCC2^ largely prevails over APC/C^Cdc20^ for both sequential and competitive inhibition models through a ‘funneling’ mechanism that is explained by the combined binding strength of the inhibitory pathway.

### Fundamental differences between sequential and competitive inhibition become apparent for high Cdc20 levels

Interestingly, for high levels of total Cdc20, when Mad and APC/C become limiting (Cdc20_total_>Mad_total_, APC/C_total_), the two models (i) and (ii) diverge. In this regime, all binding reactions involving free Cdc20 are essentially saturated, and therefore the concentrations of all species that directly bind to Cdc20 can be approximated as zero. In the sequential inhibition model, free Mad and free APC/C become negligible, and the system reduces to reaction 3, identified by the dotted box in Fig 3C. The equilibrium of this reaction depends only on the concentrations of APC/C_total_ and Mad_total_ and on the dissociation constant $K_{D}^{3}$ (S1 Text, eq 29). In particular, this means that the levels of MCC1, APC/C^MCC2^ and APC/C^Cdc20^ will stay constant, regardless of further increases in Cdc20_total_. Furthermore, if $K_{D}^{3}$ is small compared to typical protein concentrations, as it should be for efficient binding, the ratio between APC/C^Cdc20^ and APC/C^MCC2^ will be small, and APC/C^MCC2^ will dominate (Fig 3C, lower panel). Thus, this model exhibits a steady state behavior that is consistent with an active mitotic checkpoint at high Cdc20 concentrations.

In the competitive inhibition model (ii), the species that can directly bind to Cdc20, and thus are negligible when Cdc20 is in excess, are Mad, MCC1 and APC/C. Since reactions 1 and 4 become irrelevant, the system is effectively reduced to a competition of MCC2 and Cdc20 for APC/C (Fig 3D, box). In this regime, Cdc20 predominantly acts as an activator because its role as an inhibitor is limited by the saturation of Mad. Thus, when Cdc20 is in large excess, it will outcompete MCC2 and capture most of APC/C. As a consequence, the ratio between APC/C^Cdc20^ and APC/C^MCC2^ increases linearly (Fig 3D, lower panel). We can obtain explicit equations for APC/C^Cdc20^ and APC/C^MCC2^ as a function of Cdc20_total_ for the special case that the K_D_s for all complexes are the same (S1 Text, eqs 61 and 62). However, the general prevalence of APC/C^Cdc20^ at increasing Cdc20 values holds true independently of specific parameters (S1 Text, eq 54). Thus, the competitive inhibition model exhibits mitotic checkpoint deficiency for high Cdc20 levels.

### The combined model mimics the competitive inhibition model

Since the sequential (i) and the competitive inhibition model (ii) exhibit the same behavior for small Cdc20 levels, which results from Cdc20 funneling, it is not surprising that the same behavior is also present in the combined model (S1 Text, Section 3.3). More interesting is the behavior of the combined model for high levels of Cdc20, where model (i) and (ii) display a different dependency on Cdc20_total_ (Fig 3C and 3D). We observe that (iii) can be formed from the competitive inhibition model (ii) by adding reaction 3 (Fig 2). However, this addition is inconsequential in the high Cdc20 regime because MCC1 will be depleted at the expense of MCC2. Since the newly added reaction 3 is essentially inactive, the combined model reduces to the competitive inhibition model (ii), and therefore shows a dominance of APC/C^Cdc20^ at high Cdc20 concentrations (S1 Text, Section 3.3).

### Parameters for numerical analysis are chosen according to experimental results

The analytical results are useful because they allow us to understand the qualitative behavior of the models without requiring specific parameter values. Yet, these results can be obtained only under limiting assumptions. To have a more complete understanding of our models, we simulated them numerically using plausible parameter values obtained from experimental data. In this way, we also explored the region between extreme Cdc20 levels and assessed whether the investigated low and high Cdc20 regimes are physiologically relevant.

**Fig 4:**
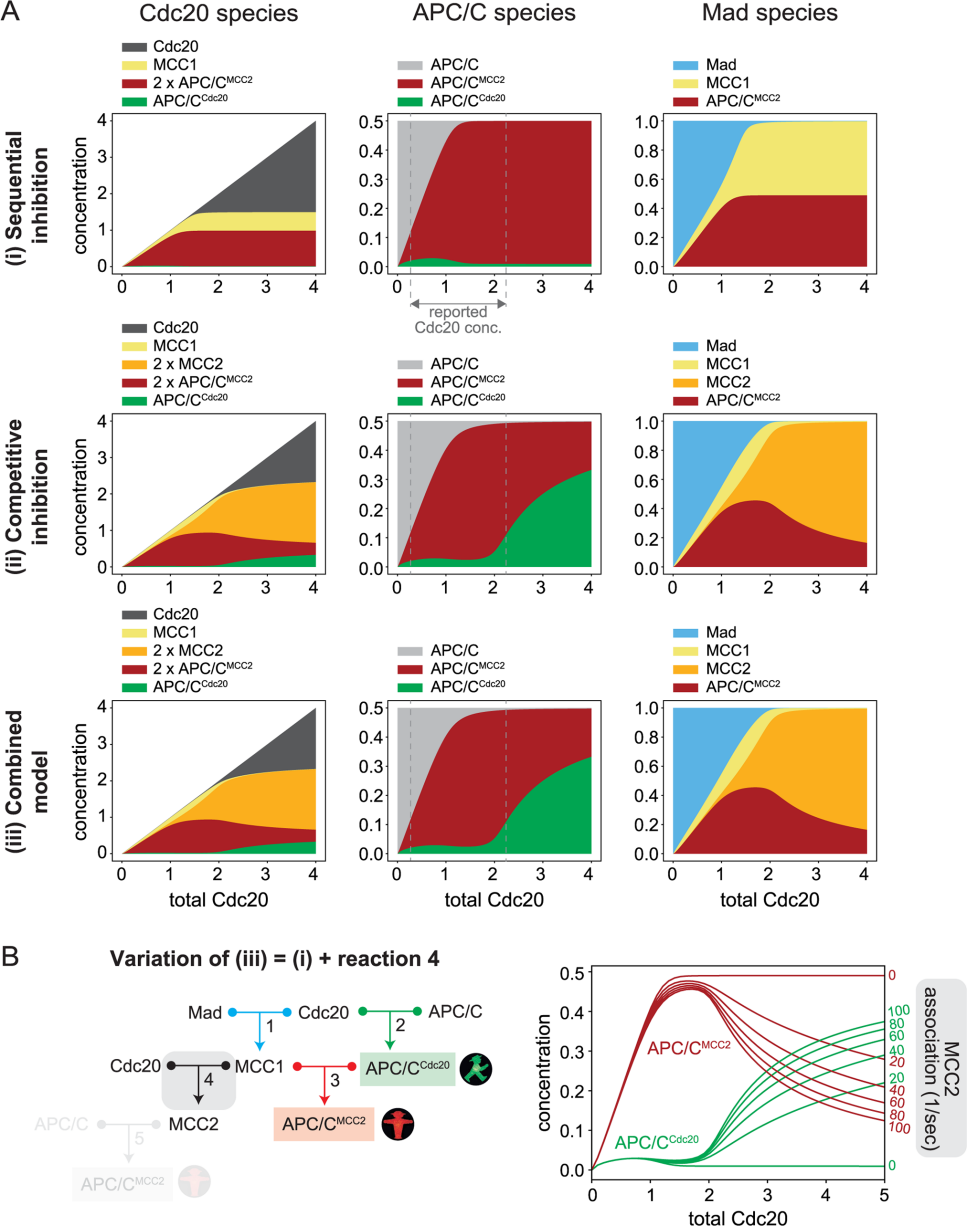
Numerical solutions for all models. **(A)** Steady state concentration of each species for varying total Cdc20 concentration and for networks (i), (ii), and (iii). All concentrations are normalized to the total concentration of Mad. APC/C is present at a Mad-normalized value of 0.5 (see S4 Fig for APC/C_total_ = 1). The molecular species are organized into three groups: those that include Cdc20, APC/C, or Mad. The plots show how these three species are distributed among different complexes. While total APC/C and total Mad remain constant, total Cdc20 increases along the x-axis. MCC2 and APC/C^MCC2^ include two molecules of Cdc20, thus their concentration is counted twice in the plots representing the Cdc20-species. The vertical white dashed lines in the panels for APC/C indicate the physiological Cdc20 range based on reported measurements. Full model descriptions in S1 Text. **(B)** Wiring diagram and simulations analogous to (A) for a variation of network (iii) including reactions 1, 2, 3 and 4, but not reaction 5 (shaded). By increasing the MCC2 association rate (reaction 4, marked in grey), the network switches from a sequential inhibition network behavior (association rate = 0) to a competitive inhibition network behavior (association rate > 0). The dissociation rate of reaction 4 remained unchanged, while the association rate was modified.

The concentrations of several checkpoint proteins have been measured [20, 37, 41, 50–56], and the kinetics of several relevant reactions have been studied *in vitro* [12–14, 50, 57–59]. Measurements of mitotic checkpoint protein concentrations vary for different molecular species across model organisms and across studies (S1 Table and S1 Fig). However, a common pattern emerges. Absolute values for what we call Mad (Mad3 and the fraction of Mad2 not bound to Mad1) are in the range of ~50 to ~200 nM. APC/C is present in lower concentrations, between ~20 and ~80 nM. Considering these results, we opted for an APC/C_total_ to Mad_total_ ratio of 0.5 for our simulations (Fig 4). For simplicity, we normalize all concentrations to that of Mad_total_.

Measurements of Cdc20 are quite variable between studies, probably partly due to the difficulty in measuring the concentration of a protein that has a high turn-over and that varies across the cell cycle [39]. They range from 0.25-times to 2.25-times that of Mad3 (S1 Table and S1 Fig). Given that we investigate the steady state behavior of our models as a function of Cdc20 levels, we cover a large range of Cdc20 concentrations, from below to above these values.

For the binding constants, we set all K_D_s to 0.01 (on the Mad_total_-normalized scale), which in absolute terms corresponds to a low nanomolar range. This value agrees with published data for binding reactions leading to MCC formation and APC/C inhibition [12, 40]. The use of identical K_D_s for all reactions serves to emphasize the differences that are due to network topology.

### Numerical simulations validate and expand the analytical solutions

For low levels of Cdc20_total_, the numerical simulations follow the analytical results for all models (Fig 4A, see S2 Fig for direct comparison) and confirm the presence of a funneling effect, resulting in an excess of APC/C^MCC2^ over APC/C^Cdc20^. As expected, the analytical approximation is particularly good when the complexes are stable (i.e., small K_D_s, compare S2A and S2B Fig, S1 Text, eq 23). For high Cdc20_total_ values, our analytical calculations show that the inactive APC/C^MCC2^ dominates in the sequential inhibition model and stays constant with increasing Cdc20_total_, while active APC/C^Cdc20^ dominates in the other two models and keeps increasing with increasing Cdc20_total_. The numerical simulations (Fig 4A) corroborate these results (see S2 Fig for direct comparison). For the sequential inhibition model, the analytical solution for large Cdc20_total_ was obtained with the assumption of identical K_D_s, although APC/C^Cdc20^ prevailing over APC/C^MCC2^ was not dependent on the specific choice of parameters. We confirmed that if we relax the assumption of identical K_D_s, our results do not change qualitatively (S3A Fig).

In general, the properties that we have described for our models are not due to a specific choice of parameter values. By randomly varying the parameter values over a realistic range, we observed that the funneling effect is a robust property of all three different networks (i.e., APC/C^MCC2^ consistently prevails over APC/C^Cdc20^ for low Cdc20 levels). Similarly, the resilience of the sequential inhibition model to high Cdc20_total_ levels and the sensitivity of the competitive inhibition and combined models to high Cdc20_total_ levels are robust properties (S3B Fig).

The analytical results predicted that the behavior of the combined model (iii) is virtually indistinguishable from that of the competitive inhibition model (S1 Text, Section 3.3), which is confirmed by the numerical simulations (Fig 4A). Both models (ii) and (iii) differ from the sequential inhibition model in that they allow the formation of free MCC2. To better understand the role of this species, we created a modification of the combined network, where we included MCC2 formation (reaction 4), but did not allow free MCC2 to interact with the APC/C (reaction 5, Fig 4B). Even though MCC2 formation is now a ‘dead end’, our simulations show that the prevalence of APC/C^Cdc20^ for high Cdc20 levels is also present in this network (Fig 4B). The reason is that MCC1 is depleted at the expense of MCC2 and cannot inhibit APC^Cdc20^. Thus, simply allowing for the formation of free MCC2 is enough to turn a system capable of buffering extra Cdc20 levels into a system that is vulnerable to Cdc20 overexpression (Fig 4B).

In summary, our numerical simulations confirm the validity of our analytical results.

### Models allowing formation of free MCC2 can be checkpoint proficient

Interestingly, numerical simulations allow us to explore intermediate Cdc20 levels, where Cdc20 can neither be considered limiting nor in excess (Fig 4A). All models show low APC/C^Cdc20^ concentrations (i.e. mitotic checkpoint proficiency) at Cdc20_total_ levels below 2 on the Mad_total_-normalized scale. In the competitive inhibition model (ii) and the combined model (iii), APC/C^Cdc20^ starts to increase at the expense of APC/C^MCC2^ when Cdc20_total_ is larger than 2. This transition is intuitively understandable from the fact that two Cdc20 molecules are captured for each molecule of Mad during MCC2 formation, and APC/C^Cdc20^ starts to increase when Mad is saturated (Fig 4A). Different studies, even in the same organism, vary widely for the reported Cdc20 concentrations (S1 Table, S1 Fig). These physiological values of Cdc20 are between 0.25 and 2.25 on our Mad-normalized scale (S1 Table), and therefore fall in the region in which APC/C^Cdc20^ is a small fraction of total APC/C (Fig 4), i.e. a condition of checkpoint proficiency.

Our results were obtained in the approximation where Mad2 and Mad3/Bub3 are lumped together in the Mad variable, and APC/C_total_ concentration is half of Mad_total_ concentration. Both approximations can be relaxed. If we explicitly introduce sequential Mad2 and Mad3/BubR1 binding, the behavior of the three models does not change qualitatively (S3C Fig). Likewise, using a 1 to 1 ratio between Mad_total_ and APC/C_total_ yields qualitatively similar results (S4 Fig).

Thus, physiologically observed Cdc20 concentrations for all models are consistent with checkpoint proficiency.

### The sensitivity of the mitotic checkpoint network can be explained by the funneling effect

**Fig 5:**
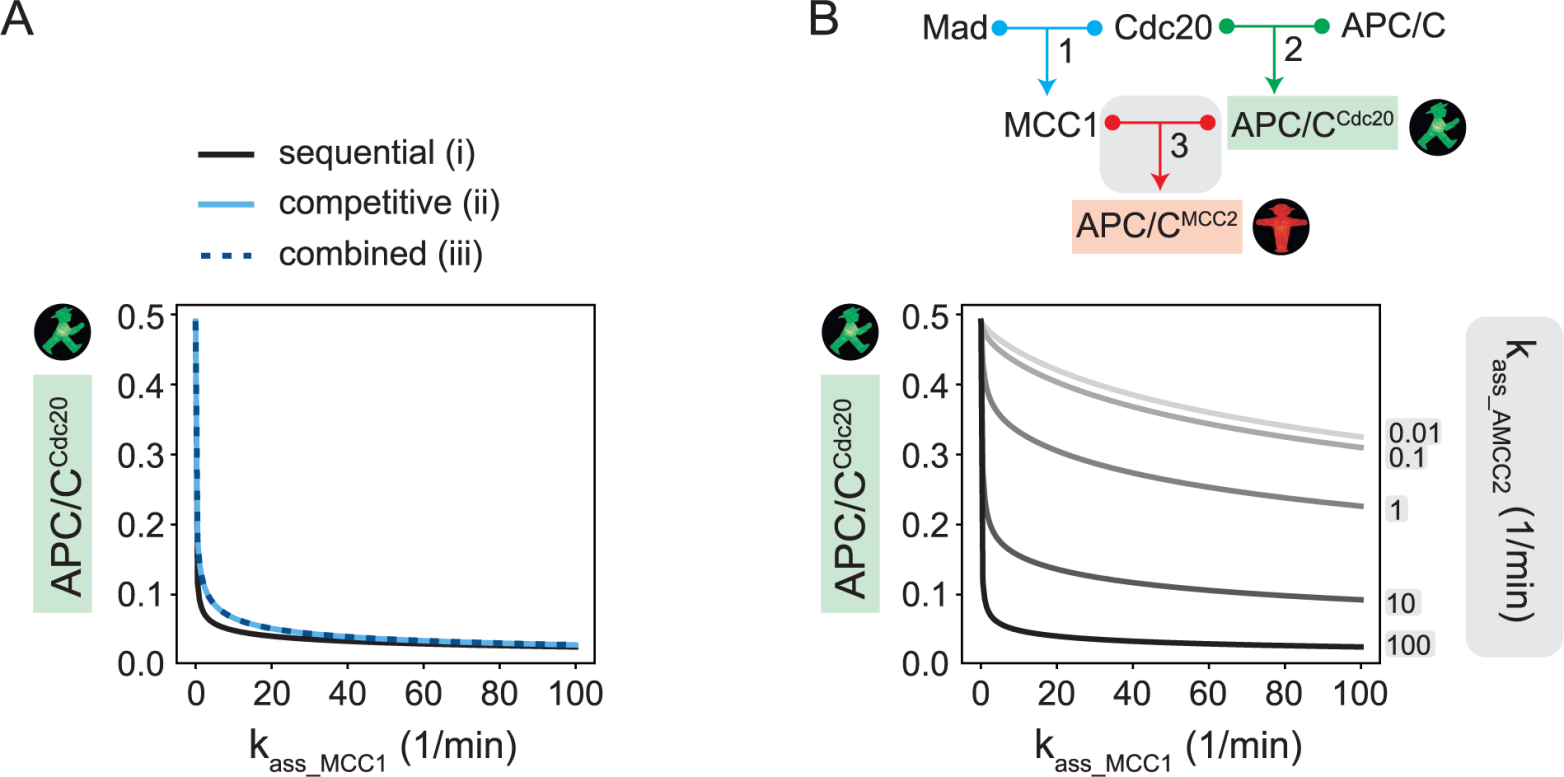
Sensitivity of the mitotic checkpoint response. **(A)** Steady state values for APC/C^Cdc20^ (normalized to total Mad) when varying the rate of MCC1 formation for models (i), (ii), and (iii). It is reasonable to assume that the association rate for an active checkpoint lies between 5 and 500 (in units of 1/min*[total Mad]) [14, 42]. **(B)** By decreasing the association rate k_ass_AMCC2_ of reaction 3 in the sequential inhibition model (top), the funneling effect is weakened, as shown by checkpoint response curves for different values of k_ass_AMCC2_ (bottom). The same effect can be seen in the competitive and combined models, which share the same funnelling effect with the sequential inhibition model.

To avoid aneuploidies, the mitotic checkpoint must respond to even a single unattached kinetochore. We asked whether this kind of sensitivity can be explained by our models. A proxy for the number of unattached kinetochores is the reaction rate that controls Mad2/Cdc20 binding, k_ass_MCC1_, which is the rate-limiting step of the pathway. To analyze the sensitivity of the system, we varied this parameter and evaluated the APC/C^Cdc20^ levels at steady state. Our results show that APC/C^Cdc20^ levels are low for all three models until k_ass_MCC1_ approaches extremely small values of around 0.001 of its default value (Fig 5A). At this point, APC/C^Cdc20^ increases strongly and nonlinearly. Hence, all three models show highly sensitive behavior, i.e. they are likely to respond appropriately to variations in the number of unattached kinetochores.

Funneling of Cdc20 into inhibitory complexes contributes to this property of the model. We described above that APC/C^MCC2^ dominates over APC/C^Cdc20^ when APC/C^Cdc20^ > *k* with $k=\frac{{APC/C}_{total}{∙K}_{D}^{1}∙K_{D}^{3}}{{Mad}_{total}{∙K}_{D}^{2}}$ (Fig 3A; eq 22—and similarly eq 44—in S1 Text). Since $K_{D}^{1}$ is proportional to 1/k_ass_MCC1_, *k* is proportional to 1/ k_ass_MCC1_. Therefore, *k* does not increase significantly until k_ass_MCC1_ approaches very small values. Thus, the strong sensitivity of the checkpoint response in our models can be explained by the funneling effect. To confirm this interpretation, we investigated the checkpoint response curve for weakened funneling, i.e. for lower values of the downstream association rate that controls the production of APC/C^MCC2^ (Fig 5B). Apart from leading to generally higher APC/C^Cdc20^ levels, weaker funneling decreases the sensitivity of the checkpoint response, i.e. APC/C^Cdc20^ levels increase more gradually as the Mad2/Cdc20 association rate is decreased (Fig 5B).

In conclusion, our results suggest that the funneling effect underlies the ability of the mitotic checkpoint network to mount a strong inhibitory response which is robust to large variations of the Mad2/Cdc20 association rate, while exhibiting sensitivity when this rate becomes very small.

## Discussion

In this paper, we theoretically analyzed the behavior of possible mitotic checkpoint assembly networks. This aspect has not been addressed by previous checkpoint models. Early models focused on the important aspect that robust inhibition and fast reactivation time are competing design constraints [7, 9]. Later work has focused on the role of the mitotic checkpoint in ensuring an irreversible transition into anaphase [5], and on the role of the mitotic spindle in checkpoint signaling [3, 4]. Many of these models greatly simplify molecular details of mitotic checkpoint signaling. On the other hand, very comprehensive models of the mitotic checkpoint network have been produced [8], but since they include a large number of mostly unmeasured parameters, they cannot be used to unambiguously answer specific questions about checkpoint signaling. Here, we chose to focus on alternative wirings for APC/C^MCC2^ assembly that cannot be ruled out based on available experimental data. Furthermore, we limited our analysis to the steady state behavior of the SAC network, which enabled analytical approaches that yield results that are not dependent on precise parameter values since those are always difficult to ascertain.

In particular, we analyzed three networks: one where APC/C^Cdc20^ is the precursor for APC/C^MCC2^ [(i) sequential inhibition], a second where Cdc20 and MCC2 compete for APC/C [(ii) competitive inhibition], and finally a third which is the combination of the first two [(iii) combined model]. We showed that all three models displayed the same behavior when Cdc20 is limiting compared to APC/C and Mad. However, their behavior differs radically when Cdc20 is in excess. While the sequential inhibition model (i) can buffer extra Cdc20, competitive inhibition (ii) and combined model (iii) fail to do so. Interestingly, the very presence of free MCC2 suffices to turn the robust sequential model into a system sensitive to Cdc20 overexpression.

The latter result is not completely unexpected. Based on intuitive assumptions, it was previously suggested that the formation of free MCC2, i.e. unbound to APC/C, is unlikely, because it would not be compatible with a robust checkpoint response [28]. The reasoning was that MCC1 must have a higher affinity for APC/C^Cdc20^ than for free Cdc20 in order to efficiently avoid APC/C^Cdc20^ formation. Our analysis confirms that allowing free MCC2 formation creates sensitivity to high levels of Cdc20. Noticeably, however, our data show that free MCC2 formation is compatible with a mitotic checkpoint response for a large range of measured Cdc20 levels in physiological conditions (Fig 4). This is due to the previously unrecognized effect of ‘funneling’ Cdc20 into inhibitory complexes. Funneling results from the fact that three binding steps are required to produce APC/C^MCC2^, whereas only one step is needed to form APC/C^Cdc20^. Even using perfectly symmetrical parameter settings (same K_D_s for all reactions and same concentrations for all species), this topological difference between the activating and inactivating branches of the mitotic checkpoint pathway results in Cdc20 accumulation in the inhibitory pathway (S4 Fig). Our analytical results show that this is true as long as total APC/C does not exceed total Mad and the dissociation constants are small compared to the concentrations of reactants. Both conditions are reasonable, and in agreement with experimental data (S1 Table, [12, 40]). In our main analysis, we did not explicitly represent all reactions leading to MCC assembly for reasons of simplicity. It should be noted that the funneling effect becomes even stronger if we explicitly model MCC formation as the physiologic two-step process of Mad2 binding, followed by Mad3 binding (S1 Text, Section 3.4, S3B Fig).

What kind of inhibition is present in living cells: competitive, sequential or mixed? The currently available information is not enough to favor or discard a particular model. It is also possible that different organisms use different solutions. We have shown that the qualitative behavior at high Cdc20 concentrations depends on the value of one single parameter, the stability of MCC2. If two organisms have evolved different stabilities for MCC2, they will display different mitotic checkpoint properties for high Cdc20 concentrations.

Our theoretical results have interesting translational implications. Cdc20 is overexpressed in a variety of cancer cells [60–70]. Whether Cdc20 overexpression is a primary driver or a secondary effect of carcinogenesis is not clear. Yet, it is known that the mitotic checkpoint is typically functional in cancer cells [44, 71–73]. We propose that the potential weakness coming with Cdc20 overexpression could be exploited to specifically target these cells, while not targeting healthy cells that have endogenous levels of Cdc20. The level of overexpression in cancer cells compared to normal cells of the same tissue ranges from 2–50 times for mRNA and 2–5 times for protein levels [61, 62, 64, 68]. According to our analysis, these levels imply that free MCC2 is either not formed at all or highly unstable in these cancer cells in order to allow checkpoint proficiency at higher Cdc20 concentrations. Stabilization of the MCC2 therefore may provide a mechanism to selectively kill these cells by impairing the mitotic checkpoint.

It is important to remark that we have only analyzed the steady state behavior of the different networks. While this analysis did not find strong differences between the networks at Cdc20 levels that are in the same range or lower than Mad, we cannot exclude that the transient dynamics of these networks differ. To investigate this possibility, more detailed information about the mechanisms of Cdc20 production and degradation are required. This does not only include the mechanisms of APC/C-mediated Cdc20 degradation while the checkpoint is active [39, 74], but also potential regulatory mechanisms of Cdc20 synthesis, post-translational regulation, and APC/C-independent degradation [75–78]. Our fragmentary knowledge on these processes is currently the biggest impediment in understanding the dynamical behavior of the mitotic checkpoint network.

## Methods

Our models are straightforward translations of the wiring diagrams shown in Fig 2 into ordinary differential equations using mass action kinetics. The equations of each model are described in S1 Text. All numerical simulations were carried out using the Python package SloppyCell and custom written Python functions. Detailed information about construction and analysis of the models and about parameter choice can be found in S1 Text.

## Supporting information

S1 Text Description of the mathematical models, and analytical derivations. (PDF)

S1 Table Reported concentrations of Mad1, Mad2, Mad3, APC/C, and Cdc20 in different model organisms. We omitted studies that reported: (i) Mad1 values higher than Mad2 (not leaving any free Mad2), (ii) values that could not easily be converted to molar concentrations, or (iii) stoichiometries that were widely different from other studies. Both absolute (abs.) and relative (rel.) amounts are shown. Relative amounts are normalized to Mad3. Values of free Mad2 are calculated as Mad2 − Mad1. References can be found in S1 Text.
(PDF)

S1 Fig Data from S1 Table, normalized to the value of Mad3 in each study. (TIF)

S2 Fig Comparison between the analytical solutions for the simplified models (dashed lines) and the numerical solution for the full models (solid lines, same as Fig 4A) for K_D_ = 0.01 (A) and K_D_ = 0.1 (B) on the Mad-normalized scale. Analytical solutions for low Cdc20 correspond to eqs 23–26 (sequential inhibition) and eqs 45–49 (competitive inhibition and combined model); analytical solutions for high Cdc20 are obtained from eqs 29–31 (sequential inhibition) and eqs 61–63 (competitive inhibition and combined model) in S1 Text.
(TIF)

S3 Fig Numerical simulations under relaxed assumptions. **(A)** Numerical simulations for the steady state concentrations of APC/C^MCC2^ and APC/C^Cdc20^ for different total Cdc20 concentrations in the competitive inhibition model (ii), when assuming either the same or different K_D_s for APC/C^Cdc20^ and APC/C^MCC2^ formation (reactions 2 and 5, respectively). **(B)** Robustness of model behavior under parameter variations. The ratio of APC/C^Cd20^ to APC/C^MCC2^ at Cdc20_total_ = 1 and Cdc20_total_ = 4 was evaluated for 1000 randomly generated parameter sets. In each set, parameters were independently drawn from a log-normal distribution set-value times 10^(N(0,0.1))^ that roughly varies between 0.5 and 2 times the set-value. **(C)** Numerical simulations for the steady state concentrations of APC/C^MCC2^ and APC/C^Cdc20^ for different total Cdc20 concentrations in all three models, either assuming a combined species Mad, which combines both Mad2 and Mad3 (solid lines), or assuming sequential Mad2 and Mad3 binding to Cdc20 (dashed lines).
(TIF)

S4 Fig Numerical simulations for the steady state concentration of each species, dependent on the total Cdc20 concentration for each of the networks (i), (ii), and (iii); similar to Fig 4A, except that total Mad and total APC/C concentrations are assumed to be identical (Mad = APC/C = 1). The vertical white dashed lines in the panels for APC/C indicate the physiological Cdc20 range based on reported measurements.
(TIF)
