# Supplementary material for: Implications of alternative routes to APC/C inhibition by the mitotic checkpoint complex
Source: PLoS Comput Biol. 2018 Sep 10;14(9):e1006449. doi: 10.1371/journal.pcbi.1006449 (PMC6157902; doi:10.1371/journal.pcbi.1006449)
Supplement: S1 Text — (PDF) [file pcbi.1006449.s001.pdf]

An *in-silico* analysis of inhibitory logics in the mitotic checkpoint network — Supplementary Information

Fridolin Gross, Paolo Bonaiuti, Silke Hauf and Andrea Ciliberto

Contents

1 Modeling Approach **1**

2 Model Parameters **2**

3 Analytical Results **3**
3.1 Sequential Inhibition Model . . . 3
3.2 Competitive Inhibition Model . . . 6
3.3 Combined Model . . . 10
3.4 Model with Mad3 as a separate species . . . 11

1 Modeling Approach

In the following we give more detailed information on the modeling approach used in the article, and provide some analytical results in support of the generality of our conclusions.

Our models are straightforward translations of the wiring diagrams shown in Fig 2 into ordinary differential equations using mass action kinetics. All binding reactions are assumed to be reversible. For the sake of readability we use the following abbreviations:

A	free APC/C
C	free Cdc20
M	free Mad2, Mad3
A_{total}	total APC/C
C_{total}	total Cdc20
M_{total}	total Mad2, Mad3
AC	APC/C$^{\text{Cdc20}}$
$MCC1$	MCC with one Cdc20 molecule
$MCC2$	MCC with two Cdc20 molecules
$AMCC2$	APC/C$^{\text{MCC2}}$

Furthermore, we use the following notation to refer to the net association and dissociation reaction of two species X and Y that can form a complex Z:

$$R_{X:Y\rightleftarrows Z} = k^{+}_{X:Y\rightleftarrows Z}[X]\cdot[Y] - k^{-}_{X:Y\rightleftarrows Z}[Z]\,, \tag{1}$$

where $[X]$ stands for the concentration of species X. To indicate the steady state level of X, we use the notation $[\hat{X}]$.

The dissociation constant is defined as

$$K_D^{X:Y} = \frac{k^{-}_{X:Y\rightleftarrows Z}}{k^{+}_{X:Y\rightleftarrows Z}} \quad , \tag{2}$$

For some derivations we assume that the rate is approximately the same for all reactions. In that case we will simply refer to it as K_D. We write down equations only for the concentrations of complexes. The concentrations of free A, M, and C can then be obtained from conservation relations that are justified by the observation that all checkpoint proteins and APC/C are stable and that total Cdc20 is at steady state.

All numerical simulations were carried out using the Python package "SloppyCell" [S1, S2] and custom written Python functions.

2 Model Parameters

Table S1 lists experimental measurements of the relevant species in different organisms derived from a comprehensive survey of the scientific literature. Relevant for our model are the concentrations of free Mad2 (not bound to Mad1), Mad3, APC/C, and Cdc20. With very few exceptions, these concentrations do not differ by more than a factor of five. Different studies come to very different conclusions, illustrating the difficulty of accurately determining absolute values. As a general scheme, however, total levels of APC/C are typically lower than Mad2/3 and Cdc20 (see also S1 Fig). For the simulations we further assumed that all dissociation constants are small (i.e. strong binding) and have the same value. Specifically, we used:

$$[C_{\text{total}}] = 1\,, \tag{3}$$

$$[M_{\text{total}}] = 1\,, \tag{4}$$

$$[A_{\text{total}}] = 0.5\,, \tag{5}$$

$$K_D = 0.01\,. \tag{6}$$

in dimensionless units (i.e. normalized to the total amount of Mad levels in wild type).

3 Analytical Results

3.1 Sequential Inhibition Model

Equations

The wiring diagram in Fig 2 (i) corresponds to the following set of equations:

$$\frac{d[AC]}{dt} = R_{A:C \rightleftarrows AC} - R_{AC:MCC1 \rightleftarrows AMCC2} \,, \tag{7}$$

$$\frac{d[MCC1]}{dt} = R_{M:C \rightleftarrows MCC1} - R_{AC:MCC1 \rightleftarrows AMCC2} \,, \tag{8}$$

$$\frac{d[AMCC2]}{dt} = R_{AC:MCC1 \rightleftarrows AMCC2} \,. \tag{9}$$

Furthermore, we have the following conservation relations:

$$[A_{\text{total}}] = [A] + [AC] + [AMCC2] \,, \tag{10}$$

$$[M_{\text{total}}] = [M] + [MCC1] + [AMCC2] \,, \tag{11}$$

$$[C_{\text{total}}] = [C] + [AC] + [MCC1] + 2 \cdot [AMCC2] \,. \tag{12}$$

Approximation for small Cdc20

In the following we will often exploit the fact that the models we consider are detailed balanced. This means that at steady state all forward reactions are individually balanced against their corresponding reverse reactions, or

$$R_{X:Y \rightleftarrows Z} = 0 \,, \tag{13}$$

which directly leads to

$$[\hat{Z}] = \frac{[\hat{X}][\hat{Y}]}{K_D^{X:Y}} \,. \tag{14}$$

For the sequential inhibition model, detailed balancing can easily be shown. Setting (9) to zero, we immediately get

$$R_{AC:MCC1 \rightleftarrows AMCC2} = 0 \,. \tag{15}$$

But then it follows from (7) and (8) that both other reactions are also detailed balanced.

Therefore, we obtain

$$[\hat{AC}] = \frac{[\hat{A}][\hat{C}]}{K_D^{A:C}} , \tag{16}$$

$$[M\hat{C}C1] = \frac{[\hat{M}][\hat{C}]}{K_D^{M:C}} , \tag{17}$$

$$[AM\hat{C}C2] = \frac{[\hat{AC}][M\hat{C}C1]}{K_D^{AC:MCC1}} = \frac{[\hat{A}][\hat{M}][\hat{C}]^2}{K_D^{A:C} K_D^{M:C} K_D^{AC:MCC1}} . \tag{18}$$

For small levels of C_{total} we can assume that most species are in their free form and therefore use the approximation

$$[\hat{A}] \approx [A_{\text{total}}] \quad \text{and} \quad [\hat{M}] \approx [M_{\text{total}}]. \tag{19}$$

Then from (16) and (17) we immediately get

$$\frac{[\hat{AC}]}{[M\hat{C}C1]} \approx \frac{[A_{\text{total}}]}{[M_{\text{total}}]} \frac{K_D^{M:C}}{K_D^{A:C}} . \tag{20}$$

Thus, in this regime the relative amounts of AC and $MCC1$ are directly related to the total amounts and the corresponding binding reactions. Furthermore, using (20) we can rewrite (18) as

$$[AM\hat{C}C2] = \frac{[M_{\text{total}}][\hat{AC}]^2 K_D^{A:C}}{[A_{\text{total}}] K_D^{M:C} K_D^{AC:MCC1}} , \tag{21}$$

or

$$[AM\hat{C}C2] \approx \frac{1}{k}[\hat{AC}]^2 \quad \text{with} \quad k = \frac{[A_{\text{total}}]}{[M_{\text{total}}]} \frac{K_D^{M:C} K_D^{AC:MCC1}}{K_D^{A:C}} . \tag{22}$$

The quadratic dependence means that $AMCC2$ will dominate for [AC] larger than k. But k is a small number if binding is generally strong and/or $[M_{\text{total}}] > [A_{\text{total}}]$. Note that k is small even if all reactions have the same binding strength and and $[M_{\text{total}}] = [A_{\text{total}}]$. This is because there are three binding reactions leading to the inhibited, but only one reaction leading to the active species. Mathematically this is reflected by the product of two K_Ds in the numerator of (22). Together this explains the "funneling" effect that we observe for small levels of Cdc20.

We can find approximate solutions for the species concentrations as a function of $[C_{\text{total}}]$ under the assumption of strong binding (i.e. $K_D \ll A_{\text{total}}, M_{\text{total}}$). In this case $[AM\hat{C}C2]$ dominates the sum in (12). In particular, we have

$$[AM\hat{C}C2] \to \frac{[C_{\text{total}}]}{2} \quad \text{for} \quad K_D \to 0 , \tag{23}$$

which explains the initially approximately linear behavior of APC/C$^{\mathrm{MCC2}}$ in S2 Fig. Based on this first order approximation, we can use Equations (16)–(18) to derive expressions for the other species as well:

$$[\hat{C}] \approx \sqrt{\frac{K_D^{A:C} K_D^{M:C} K_D^{AC:MCC1}}{2[A_{\text{total}}][M_{\text{total}}]}} \sqrt{[C_{\text{total}}]} \,, \tag{24}$$

$$[\hat{AC}] \approx \sqrt{\frac{1}{2} \frac{[A_{\text{total}}]}{[M_{\text{total}}]} \frac{K_D^{M:C} K_D^{AC:MCC1}}{K_D^{A:C}}} \sqrt{[C_{\text{total}}]} \,, \tag{25}$$

$$[M\hat{C}C1] \approx \sqrt{\frac{1}{2} \frac{[M_{\text{total}}]}{[A_{\text{total}}]} \frac{K_D^{A:C} K_D^{AC:MCC1}}{K_D^{M:C}}} \sqrt{[C_{\text{total}}]} \,. \tag{26}$$

These expressions indicate how the steady state concentrations for small levels of Cdc20 depend on parameters and concentrations. In particular, they explain the approximately linear dependence of APC/C$^{\mathrm{MCC2}}$ and the square root dependence of the other species. The approximations are in good quantitative agreement with the simulations for small values of K_D. But even for larger values they provide a qualitative understanding of the behavior of the model (S2 Fig).

Approximation for large Cdc20

If C_{total} is large, then every free molecule of M or A will quickly bind to a free molecule of C. With the approximation that this binding is instantaneous and $M \approx A \approx 0$, we can directly calculate the steady state values for the remaining species. For this it is sufficient to look at the three species $MCC1$, AC, and $AMCC2$, and the only reaction left to be considered is

$$\frac{d[AMCC2]}{dt} = R_{AC:MCC1 \rightleftarrows AMCC2} \,, \tag{27}$$

because the other two species are then determined by the conservation relations. At steady state we have

$$[AM\hat{C}C2] = \frac{[\hat{AC}] \cdot [M\hat{C}C1]}{K_D^{AC:MCC1}} = \frac{([A_{\text{total}}] - [AM\hat{C}C2])([M_{\text{total}}] - [AM\hat{C}C2])}{K_D^{AC:MCC1}} \,.$$

From this we get

$$([A_{\text{total}}] - [AM\hat{C}C2])([M_{\text{total}}] - [AM\hat{C}C2]) - K_D^{AC:MCC1} \cdot [AM\hat{C}C2] = 0 \,, \tag{28}$$

which is a quadratic equation in $[AM\hat{C}C2]$ whose solutions are

$$[AM\hat{C}C2] = \frac{[A_{\text{total}}] + [M_{\text{total}}] + K_D^{AC:MCC1}}{2} \pm \sqrt{\left(\frac{[A_{\text{total}}] + [M_{\text{total}}] + K_D^{AC:MCC1}}{2}\right)^2 - [A_{\text{total}}][M_{\text{total}}]} \,. \tag{29}$$

Only the "−" solution ensures that $[AM\hat{C}C2] \leq [M_{\text{total}}], [A_{\text{total}}]$. Expressions for the other species can be directly inferred, using

$$[A\hat{C}] = [A_{\text{total}}] - [AM\hat{C}C2]\,, \tag{30}$$

$$[M\hat{C}C1] = [M_{\text{total}}] - [AM\hat{C}C2]\,. \tag{31}$$

The relative amounts of $[AM\hat{C}C2]$ and $[A\hat{C}]$ mainly depend on the strength of AC binding to $MCC1$. For weak binding ($K_D^{AC:MCC1} \gg [M_{\text{total}}], [A_{\text{total}}]$) we get $AMCC2 \approx 0$, while for strong binding we get $[A\hat{C}] \approx 0$. In general, both species co-exist, and their levels are insensitive to changes in C_{total} levels.

In S2 Fig (i) the simulated steady state concentrations for the sequential model are shown together with the analytical approximations for small and large Cdc20.

3.2 Competitive Inhibition Model

Equations:

The competitive inhibition model includes the additional species $MCC2$ which is formed when $MCC1$ binds an additional molecule of C. The inhibited species $AMCC2$ is in this case formed by $MCC2$ binding to a free molecule of A. We therefore have to consider four equations:

$$\frac{d[AC]}{dt} = R_{A:C \rightleftarrows AC}\,, \tag{32}$$

$$\frac{d[MCC1]}{dt} = R_{M:C \rightleftarrows MCC1} - R_{MCC1:C \rightleftarrows MCC2}\,, \tag{33}$$

$$\frac{d[MCC2]}{dt} = R_{MCC1:C \rightleftarrows MCC2} - R_{A:MCC2 \rightleftarrows AMCC2}\,, \tag{34}$$

$$\frac{d[AMCC2]}{dt} = R_{A:MCC2 \rightleftarrows AMCC2}\,, \tag{35}$$

together with the following conservation relations:

$$[A_{\text{total}}] = [A] + [AC] + [AMCC2]\,, \tag{36}$$

$$[M_{\text{total}}] = [M] + [MCC1] + [MCC2] + [AMCC2]\,, \tag{37}$$

$$[C_{\text{total}}] = [C] + [AC] + [MCC1] + 2 \cdot ([MCC2] + [AMCC2])\,. \tag{38}$$

Approximation for small Cdc20

In the same way as before we can show that detailed balancing holds, and we get at steady state

$$[\hat{AC}] = \frac{[\hat{A}][\hat{C}]}{K_D^{A:C}}\,, \tag{39}$$

$$[M\hat{C}C1] = \frac{[\hat{M}][\hat{C}]}{K_D^{M:C}}\,, \tag{40}$$

$$[M\hat{C}C2] = \frac{[M\hat{C}C1][\hat{C}]}{K_D^{MCC1:C}} = \frac{[\hat{M}][\hat{C}]^2}{K_D^{M:C}K_D^{MCC1:C}}\,, \tag{41}$$

$$[AM\hat{C}C2] = \frac{[\hat{A}][M\hat{C}C2]}{K_D^{A:MCC2}} = \frac{[\hat{A}][\hat{M}][\hat{C}]^2}{K_D^{M:C}K_D^{MCC1:C}K_D^{A:MCC2}}\,. \tag{42}$$

Equations (39) and (40) are identical to (16) and (17), and the only difference between (42) and (18) is one of the dissociation constants. Moreover, for strong binding $[M\hat{C}C2]$ is very small compared to the other species because, for example, from (41) and (42) we get

$$\frac{M\hat{C}C2}{AM\hat{C}C2} \approx \frac{K_D^{AC:MCC2}}{[A_{\text{total}}]}\,. \tag{43}$$

So the model effectively reduces to the same equations as the sequential inhibition model. In particular we get the equivalent of (22) by rewriting (42) using (39):

$$[AM\hat{C}C2] \approx \frac{1}{k}[\hat{AC}]^2 \quad \text{with} \quad k = \frac{[A_{\text{total}}]}{[M_{\text{total}}]}\frac{K_D^{M:C}K_D^{MCC1:C}K_D^{A:MCC2}}{(K_D^{A:C})^2}\,. \tag{44}$$

This explains why for small levels of Cdc20 the steady state behavior is basically the same for sequential and competitive inhibition given equivalent choice of parameters. Again, k is a small number provided that binding is strong and A_{total} does not exceed M_{total}. Moreover, we see that the funneling effect is indifferent to the order in which the complexes are formed. This is a straightforward consequence of mass action kinetics and detailed balancing.

Analogously to the case of sequential inhibition, $[AMCC2]$ dominates (38) for $K_D \to 0$, so we can

derive the following approximations:

$$[A\hat{MCC}2] \approx \frac{[C_{\text{total}}]}{2}\,, \tag{45}$$

$$[\hat{C}] \approx \sqrt{\frac{K_D^{M:C} K_D^{MCC1:C} K_D^{A:MCC2}}{2[A_{\text{total}}][M_{\text{total}}]}}\sqrt{[C_{\text{total}}]}\,, \tag{46}$$

$$[\hat{AC}] \approx \sqrt{\frac{1}{2}\frac{[A_{\text{total}}]}{[M_{\text{total}}]}\frac{K_D^{M:C} K_D^{MCC1:C} K_D^{A:MCC2}}{(K_D^{A:C})^2}}\sqrt{[C_{\text{total}}]}\,, \tag{47}$$

$$[M\hat{C}C1] \approx \sqrt{\frac{1}{2}\frac{[M_{\text{total}}]}{[A_{\text{total}}]}\frac{K_D^{MCC1:C} K_D^{A:MCC2}}{K_D^{M:C}}}\sqrt{[C_{\text{total}}]}\,, \tag{48}$$

$$[M\hat{C}C2] \approx \frac{K_D^{A:MCC2}}{2[A_{\text{total}}]}[C_{\text{total}}]\,. \tag{49}$$

Again we reproduce the approximately linear behavior of APC/C$^{\text{AMCC2}}$ and the square root behavior of the other species (with the exception for free $MCC2$, which increases linearly, but with a very small slope).

Approximation for large Cdc20

For high levels of C_{total}, we can assume that

$$[M_{\text{total}}] \approx [AMCC2] + [MCC2]\,, \tag{50}$$

$$[A_{\text{total}}] \approx [AMCC2] + [AC]\,, \tag{51}$$

meaning that all species that bind to free C (i.e. A, M, and MCC) are approximately zero. With the help of the conservation relation (38) we can then immediately derive

$$[C_{\text{total}}] \approx 2 \cdot [M_{\text{total}}] + [AC] + [C]\,. \tag{52}$$

This means that the system effectively reduces to a simple competition model where the inhibitor $MCC2$ competes with C for free A.

It can be easily shown that the active species will always outcompete the inactive species if levels of Cdc20 are high. First of all, note that if $[C_{\text{total}}] \to \infty$, then also $[C] \to \infty$. From detailed balancing, we then get

$$[A] = \frac{K_D^{A:C}[AC]}{[C]} < \frac{K_D^{A:C}[A_{\text{total}}]}{[C]} \to 0\,, \tag{53}$$

which entails

$$[AMCC2] = \frac{[A][MCC2]}{K_D^{A:MCC2}} < \frac{[A][M_{\text{total}}]}{K_D^{A:MCC2}} \to 0\,. \tag{54}$$

For the special case of $K_D = K_D^{A:MCC2} = K_D^{A:C}$, we can derive simple expressions for $[\hat{AC}]$ and $[AM\hat{C}C2]$ as functions of $[C_{\text{total}}]$. Given detailed balancing, Eq. (50) leads to

$$[M_{\text{total}}] \approx \frac{[\hat{A}][M\hat{C}C2]}{K_D} + [M\hat{C}C2] = \frac{K_D + [\hat{A}]}{K_D} \cdot [M\hat{C}C2]\,, \tag{55}$$

or

$$[M\hat{C}C2] \approx [M_{\text{total}}]\frac{K_D}{K_D + [\hat{A}]}\,. \tag{56}$$

From Eq. (52) we can derive an analogous expression for $[\hat{C}]$:

$$[C_{\text{total}}] - 2\cdot[M_{\text{total}}] \approx \frac{K_D + [\hat{A}]}{K_D} \cdot [\hat{C}]\,, \tag{57}$$

or

$$[\hat{C}] \approx \left([C_{\text{total}}] - 2\cdot[M_{\text{total}}]\right)\frac{K_D}{K_D + [\hat{A}]}\,. \tag{58}$$

Furthermore, rewriting (51) and afterwards substituting (56) and (58), we get

$$\begin{aligned}[A_{\text{total}}] &\approx \frac{[\hat{A}][M\hat{C}C2]}{K_D} + \frac{[\hat{A}][\hat{C}]}{K_D} \\ &\approx [\hat{A}]\cdot\left(\frac{[M_{\text{total}}]}{K_D + [\hat{A}]} + \frac{[C_{\text{total}}] - 2\cdot[M_{\text{total}}]}{K_D + [\hat{A}]}\right)\,,\end{aligned} \tag{59}$$

from which we obtain

$$\frac{[\hat{A}]}{K_D + [\hat{A}]} \approx \frac{[A_{\text{total}}]}{[C_{\text{total}}] - [M_{\text{total}}]}\,. \tag{60}$$

Combining (56), (58), and (60), we finally get

$$[AM\hat{C}C2] = \frac{[\hat{A}][M\hat{C}C2]}{K_D} \approx \frac{[A_{\text{total}}][M_{\text{total}}]}{[C_{\text{total}}] - [M_{\text{total}}]} \tag{61}$$

and

$$[\hat{AC}] = \frac{[\hat{A}][\hat{C}]}{K_D} \approx \frac{[A_{\text{total}}]([C_{\text{total}}] - 2 \cdot [M_{\text{total}}])}{[C_{\text{total}}] - [M_{\text{total}}]} \,. \tag{62}$$

Furthermore, from (50) and (61) we get

$$[M\hat{C}C2] = \frac{[M_{\text{total}}([C_{\text{total}}] - [M_{\text{total}}] - [A_{\text{total}}])]}{[C_{\text{total}}] - [M_{\text{total}}]} \,. \tag{63}$$

From (61) and (62) we obtain the simple expression

$$\frac{[\hat{AC}]}{[AM\hat{C}C2]} = \frac{[C_{\text{total}}]}{[M_{\text{total}}]} - 2 \,. \tag{64}$$

Thus, the ratio of active to inactive APC/C increases linearly with the level of Cdc20.

Note that these expressions depend only on the total amounts and not on the association/dissociation parameters (in particular, we did not use the assumption of strong binding). Most importantly, and as already shown, we will always get $AC \to A_{\text{total}}$ and $AMCC2 \to 0$ for $C_{\text{total}} \to \infty$. In other words, the competitive inhibition model always becomes checkpoint deficient for sufficiently high levels of Cdc20.

The approximations for the competitive inhibition model are shown in S2 Fig (ii).

3.3 Combined Model

Equations

The combined model includes both ways of producing the inhibited species $AMCC2$. The corresponding set of equations is

$$\frac{d[AC]}{dt} = R_{A:C \rightleftharpoons AC} - R_{AC:MCC1 \rightleftharpoons AMCC2} \,, \tag{65}$$

$$\frac{d[MCC1]}{dt} = R_{M:C \rightleftharpoons MCC1} - R_{MCC1:C \rightleftharpoons MCC2} - R_{AC:MCC1 \rightleftharpoons AMCC2} \,, \tag{66}$$

$$\frac{d[MCC2]}{dt} = R_{MCC1:C \rightleftharpoons MCC2} - R_{A:MCC2 \rightleftharpoons AMCC2} \,, \tag{67}$$

$$\frac{d[AMCC2]}{dt} = R_{A:MCC2 \rightleftharpoons AMCC2} + R_{AC:MCC1 \rightleftharpoons AMCC2} \,. \tag{68}$$

The conservation relations are the same as (36), (37), and (38). For this network the detailed balancing property does not follow directly from the equations, but requires certain restricting conditions on the rate constants. It can be shown that the condition for detailed balancing for this model is

$$K_D^{A:C} \cdot K_D^{AC:MCC1} = K_D^{MCC1:C} \cdot K_D^{A:MCC2} \tag{69}$$

(a procedure for deriving this condition can be found for instance in [S3]). For our analysis we assume that detailed balancing holds. The condition is obviously fulfilled in the special case that all K_Ds are the same.

Approximation for small Cdc20

Given detailed balancing, the derivation of steady state expressions can be carried out in the same way as in the case of the competitive inhibition model in Section 3.2. The relationship between active and inhibited species is described both by (22) and (44) because the two equations coincide when condition (69) holds. This explains why the behavior of the combined model for small Cdc20 is the same as in the other two models.

Approximation for large Cdc20

As in Section 3.2, we can assume that all reactions involving free C are saturated. In particular, we have $MCC1 \approx 0$, which means that the sequential production of the inhibitor $R_{AC:MCC1 \rightleftarrows AMCC2}$, that is added with respect to the competitive inhibition model, is negligible. As a consequence, the behavior of the combined model at saturating levels of Cdc20 is the same as the competitive inhibition model.

The approximations for the combined model are shown in S3 Fig (iii).

3.4 Model with Mad3 as a separate species

Equations:

To incorporate Mad3 as a separate species, we assume that first Mad2 ($M2$) binds to Cdc20 (C) to form Mad2:Cdc20 ($M2C$). Afterwards this complex binds to free Mad3 ($M3$) to form $MCC1$. This translates to the following equations for the competitive inhibition case:

$$\frac{d[M2C]}{dt} = R_{M2:C \rightleftarrows M2C} - R_{M2C:M3 \rightleftarrows MCC1}\,, \tag{70}$$

$$\frac{d[MCC1]}{dt} = R_{M2C:M3 \rightleftarrows MCC1} - R_{MCC1:C \rightleftarrows MCC2}\,, \tag{71}$$

and to

$$\frac{d[M2C]}{dt} = R_{M2:C \rightleftarrows M2C} - R_{M2C:M3 \rightleftarrows MCC1}\,, \tag{72}$$

$$\frac{d[MCC1]}{dt} = R_{M2C:M3 \rightleftarrows MCC1} - R_{AC:MCC1 \rightleftarrows AMCC2}\,, \tag{73}$$

for the sequential inhibition case. The equations for $[AC]$, $[MCC2]$, and $[AMCC2]$ are unchanged with respect to 3.1 and 3.2.

As before, the concentrations of the free species can be obtained from conservation relations:

$$[A_{\text{total}}] = [A] + [AC] + [AMCC2] \,, \tag{74}$$

$$[M2_{\text{total}}] = [M2] + [M2C] + [MCC1] + [MCC2] + [AMCC2] \,, \tag{75}$$

$$[M3_{\text{total}}] = [M3] + [MCC1] + [MCC2] + [AMCC2] \,, \tag{76}$$

$$[C_{\text{total}}] = [C] + [AC] + [M2C] + [MCC1] + 2 \cdot ([MCC2] + [AMCC2]) \;. \tag{77}$$

Approximation for small Cdc20

We can use the same strategy as before, based on the property of detailed balancing, to derive steady state expressions for AC and $AMCC2$. But whereas the expression for AC is unchanged:

$$[\hat{AC}] \approx \frac{[A_{\text{total}}][\hat{C}]}{K_D^{A:C}} \,, \tag{78}$$

due to the additional species we now get

$$[AM\hat{C}C2] \approx \frac{[A_{\text{total}}][M2_{\text{total}}][M3_{\text{total}}][\hat{C}]^2}{K_D^{A:C} K_D^{M2:C} K_D^{M2C:M3} K_D^{AC:MCC1}} \tag{79}$$

for sequential inhibition, and

$$[AM\hat{C}C2] \approx \frac{[A_{\text{total}}][M2_{\text{total}}][M3_{\text{total}}][\hat{C}]^2}{K_D^{M2:C} K_D^{M2C:M3} K_D^{MCC1:C} K_D^{A:MCC2}} \tag{80}$$

for competitive inhibition.

As a result, we get the following relationship between active and inhibited species:

$$[AM\hat{C}C2] \approx \frac{1}{k}[\hat{AC}]^2 \,, \tag{81}$$

where

$$k = \frac{[A_{\text{total}}]}{[M2_{\text{total}}][M3_{\text{total}}]} \frac{K_D^{M2:C} K_D^{M2C:M3} K_D^{AC:MCC1}}{K_D^{A:C}} \tag{82}$$

for sequential inhibition, and

$$k = \frac{[A_{\text{total}}]}{[M2_{\text{total}}][M3_{\text{total}}]} \frac{K_D^{M2:C} K_D^{M2C:M3} K_D^{MCC1:C} K_D^{A:MCC2}}{(K_D^{A:C})^2} \tag{83}$$

for competitive inhibition.

Comparing this to (22), the corresponding equation for the model without $M3$, we see that the constant k is now even smaller (provided that the K_Ds are smaller than M2 and M3) because there are four

reactions to build the inhibited species. This means that the funneling effect is even more pronounced in a model with Mad3 as a separate species. This can be seen in S3 Fig B.

Approximation for large Cdc20

For large levels of Cdc20 the model including Mad3 behaves very similarly to the simpler models, provided that $[M3_{\text{total}}] = [M_{\text{total}}]$, i.e. $M3$ is limiting.

For the competitive inhibition scenario, the model is approximated by exactly the same reduced network as the model in 3.2. This is because $MCC1 \approx 0$, and therefore also $M3, M2C \approx 0$ (using again detailed balancing).

In the case of sequential inhibition we are left with two remaining reactions: $R_{M2C:M3 \rightleftarrows MCC1}$ and $R_{AC:MCC1 \rightleftarrows AMCC2}$. Thus there is again an equilibrium between AC and $AMCC2$, but the levels are slightly shifted because $[MCC1] < [M3_{\text{total}}]$. Under the assumption that binding of $M2C$ to $M3$ is strong, the asymptotic levels of AC and $AMCC2$ are very close to those in 3.1, as can be seen in S3 Fig B (i).

Supplemental References

[S1] Gutenkunst RN, Atlas JC, Casey FP, Daniels BC, Kuczenski RS, Waterfall JJ, Myers CR, Sethna JP (2007) SloppyCell, `http://sloppycell.sourceforge.net/`

[S2] Myers CR, Gutenkunst RN, Sethna JP (2007) Python unleashed on systems biology. *Comput Sci Eng* **9**: 34–37

[S3] Feinberg M (1989) Necessary and Sufficient Conditions for Detailed Balancing in Mass Action Systems of Arbitrary Complexity. *Chem Eng Sci* **44**: 1819–1827

[S4] Hein MY, Hubner NC, Poser I, Cox J, Nagaraj N, Toyoda Y, Gak IA, Weisswange I, Mansfeld J, Buchholz F, Hyman AA, Mann M (2015) A Human Interactome in Three Quantitative Dimensions Organized by Stoichiometries and Abundances. *Cell* **163**: 712–723

[S5] Kulak NA, Pichler G, Paron I, Nagaraj N, Mann M (2014) Minimal, encapsulated proteomic-sample processing applied to copy-number estimation in eukaryotic cells. *Nat Methods* **11**: 319–324

[S6] Shah J, Botvinick E, Bonday Z, Furnari F, Berns M, Cleveland DW (2004) Dynamics of Centromere and Kinetochore Proteins: Implications for Checkpoint Signaling and Silencing. *Curr Biol* **14**: 942–952

[S7] Luo X, Tang Z, Xia G, Wassmann K, Matsumoto T, Rizo J, Yu H (2004) The Mad2 spindle checkpoint protein has two distinct natively folded states. *Nat Struct Mol Biol* **11**: 338–345

[S8] Fang G (2002) Checkpoint Protein BubR1 Acts Synergistically with Mad2 to Inhibit Anaphase-promoting Complex. *Mol Biol Cell* **13**: 755–766

[S9] Tang Z, Bharadwaj R, Li B, Yu H (2001) Mad2-Independent inhibition of APCCdc20 by the mitotic checkpoint protein BubR1. *Dev Cell* **1**: 227–237

[S10] Sudakin V, Chan GK, Yen TJ (2001) Checkpoint inhibition of the APC/C in HeLa cells is mediated by a complex of BUBR1, BUB3, CDC20, and MAD2. *J Cell Biol* **154**: 925–936

[S11] Chong YT, Koh JL, Friesen H, Duffy SK, Cox MJ, Moses A, Moffat J, Boone C, Andrews BJ (2015) Yeast Proteome Dynamics from Single Cell Imaging and Automated Analysis. *Cell* **161**: 1413–1424

[S12] Poddar A, Stukenberg PT, Burke DJ (2005) Two Complexes of Spindle Checkpoint Proteins Containing Cdc20 and Mad2 Assemble during Mitosis Independently of the Kinetochore in *Saccharomyces cerevisiae*. *Eukaryot Cell* **4**: 867–878

[S13] Ghaemmaghami S, Huh WK, Bower K, Howson RW, Belle A, Dephoure N, O'Shea EK, Weissman JS (2003) Global analysis of protein expression in yeast. *Nature* **425**: 737–741

[S14] Bonaiuti P, Cairoli E, Gross F, Corno A, Vernieri C, Štefl M, Cosentino Lagomarsino M, Knop M, Ciliberto A (forthcoming) Cells escape an operational mitotic checkpoint through a stochastic process. *Curr Biol*

[S15] Carpy A, Krug K, Graf S, Koch A, Popic S, Hauf S, Macek B (2014) Absolute Proteome and Phosphoproteome Dynamics during the Cell Cycle of Schizosaccharomyces pombe (Fission Yeast). *Mol Cell Proteomics* **13**: 1925–1936

[S16] Heinrich S, Geissen EM, Kamenz J, Trautmann S, Widmer C, Drewe P, Knop M, Radde N, Hasenauer J, Hauf S (2013) Determinants of robustness in spindle assembly checkpoint signalling. *Nature Cell Biol* **15**: 1328–1339

[S17] Marguerat S, Schmidt A, Codlin S, Chen W, Aebersold R, Bähler J (2012) Quantitative Analysis of Fission Yeast Transcriptomes and Proteomes in Proliferating and Quiescent Cells. *Cell* **151**: 671–683

[S18] Howell BJ, Moree B, Farrar EM, Stewart S, Fang G, Salmon ED (2004) Spindle Checkpoint Protein Dynamics at Kinetochores in Living Cells. *Curr Biol* **14**: 953–964

[S19] Wühr M, Freeman Jr RM, Presler M, Horb ME, Peshkin L, Gygi S, Kirschner MW (2015) Deep Proteomics of the Xenopus laevis Egg using an mRNA-derived Reference Database. *Curr Biol* **24**: 1467–1475
