# Supplementary material for: Implications of alternative routes to APC/C inhibition by the mitotic checkpoint complex
Source: PLoS Comput Biol. 2018 Sep 10;14(9):e1006449. doi: 10.1371/journal.pcbi.1006449 (PMC6157902; doi:10.1371/journal.pcbi.1006449)
Supplement: S1 Table — We omitted studies that reported: (i) Mad1 values higher than Mad2 (not leaving any free Mad2), (ii) values that could not easily be converted to molar concentrations, or (iii) stoichiometries that were widely different from other studies. Both absolute (abs.) and relative (rel.) amounts are shown. Relative amounts are normalized to Mad3. Values of free Mad2 are calculated as Mad2 − Mad1. References can be found in S1 Text. (PDF) [file pcbi.1006449.s002.pdf]

		Mad2		Mad1		free Mad2		Mad3		APC/C		Cdc20		ref.
**organism**	**unit**	**abs.**	**rel.**	**abs.**	**rel.**	**abs.**	**rel.**	**abs.**	**rel.**	**abs.**	**rel.**	**abs.**	**rel.**	
human (HeLa)	**nM**	202	229.5%	27	30.7%	175	198.9%	88	100%	31	35.2%	24	27.3%	[S4]
	mol./cell	264,329		72,571		191,758				35,276		18,265		[S5]
	nM	200		20		180								[S6]
	rel. ratio	4		1										[S7]
	nM	230	181.1%					127	100%			285	224.4%	[S8]
	nM	120	133.3%					90	100%	80	88.9%	100	111.1%	[S9]
	nM	400	769.2%			30	57.7%	52	100%	33	62.5%			[S10]
S. cerevisiae	mol./cell	56		54		2				59		54		[S11]
	mol./cell	9,600	300.0%					3,200	100%	375	11.7%	2,200	68.8%	[S12]
	mol./cell	1,112	35.1%	656	20.7%	456	14.4%	3,171	100%	628	19.8%			[S13]
	nM	201	300.0%					67	100%	30	44.8%			[S14]
S. pombe	mol./cell			3,855	76.9%			5,012	100%	1,495	29.8%			[S15]
	nM	154	208.1%	116	156.8%	38	51.4%	74	100%	14	18.9%			[S16]
	nM	61	138.6%	39	88.6%	22	50.0%	44	100%	20	45.5%	20	45.5%	[S16]
	mol./cell			832						341				[S17]
PtK	nM	1,041	60.0%	625	36.0%	416	24.0%	1,735	100%			867	50.0%	[S18]
X. laevis	nM	185	544.1%	127	373.5%	58	170.6%	34	100%	30	88.2%	55	161.8%	[S19]
