# Supplementary figures and images for: Implications of alternative routes to APC/C inhibition by the mitotic checkpoint complex
Source: PLoS Comput Biol. 2018 Sep 10;14(9):e1006449. doi: 10.1371/journal.pcbi.1006449 (PMC6157902; doi:10.1371/journal.pcbi.1006449)

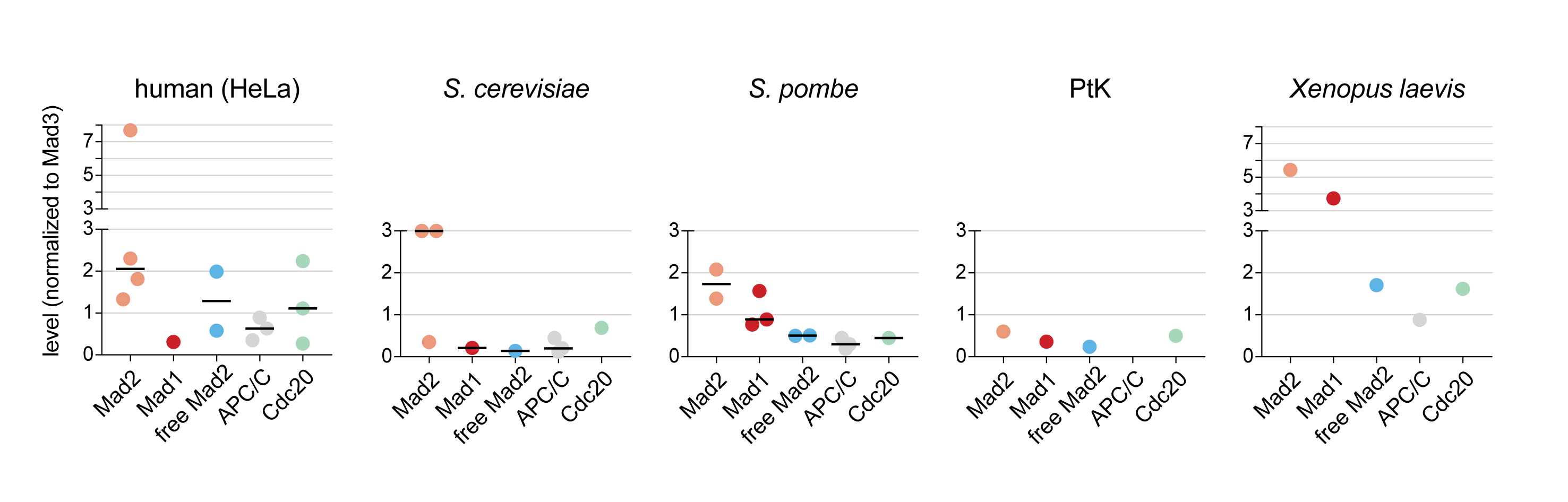

Supplement: S1 Fig — (TIF) [file pcbi.1006449.s003.tif]

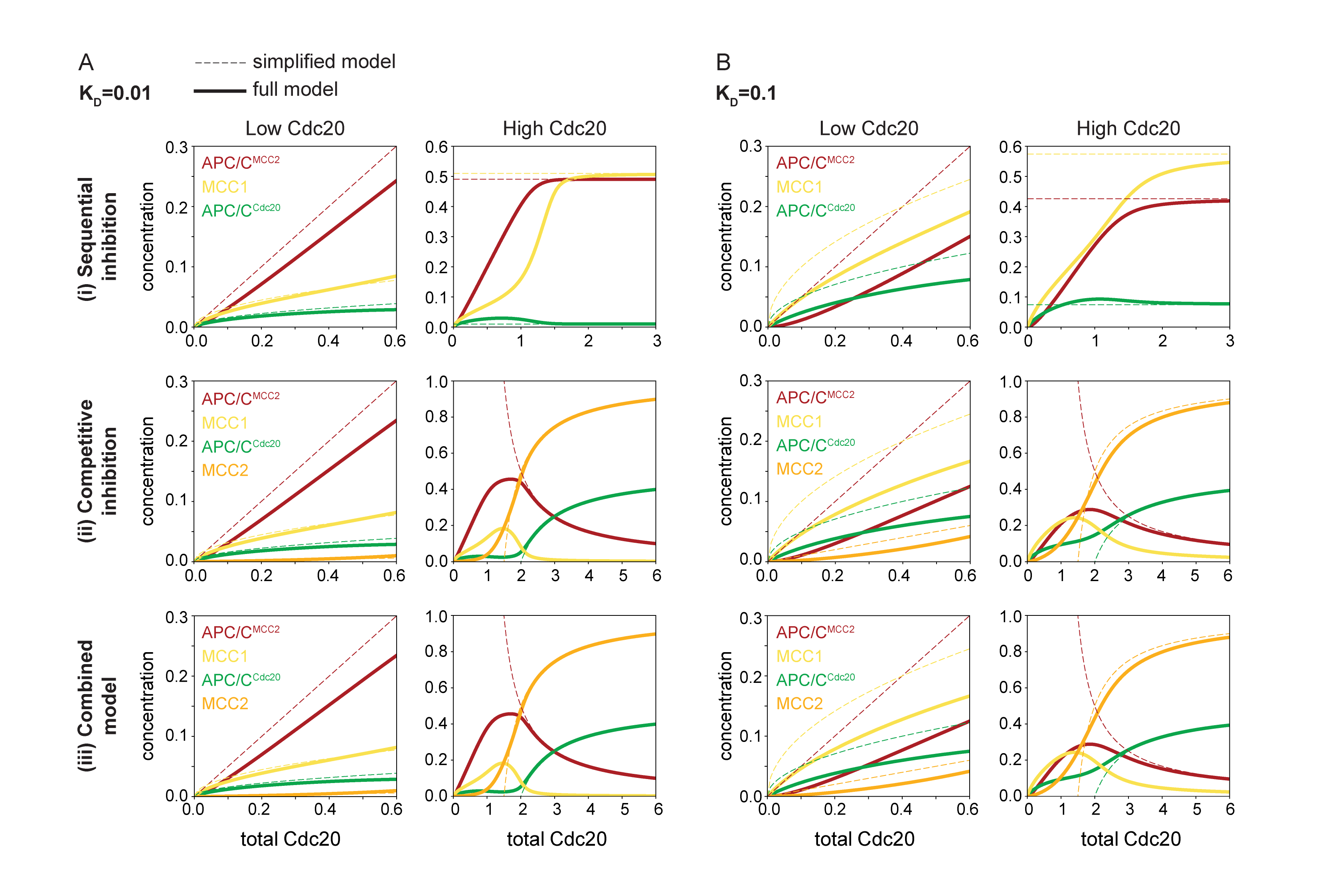

Supplement: S2 Fig — Comparison between the analytical solutions for the simplified models (dashed lines) and the numerical solution for the full models (solid lines, same as Fig 4A) for KD = 0.01 (A) and KD = 0.1 (B) on the Mad-normalized scale. Analytical solutions for low Cdc20 correspond to eqs 23–26 (sequential inhibition) and eqs 45–49 (competitive inhibition and combined model); analytical solutions for high Cdc20 are obtained from eqs 29–31 (sequential inhibition) and eqs 61–63 (competitive inhibition and combined model) in S1 Text. (TIF) [file pcbi.1006449.s004.tif]

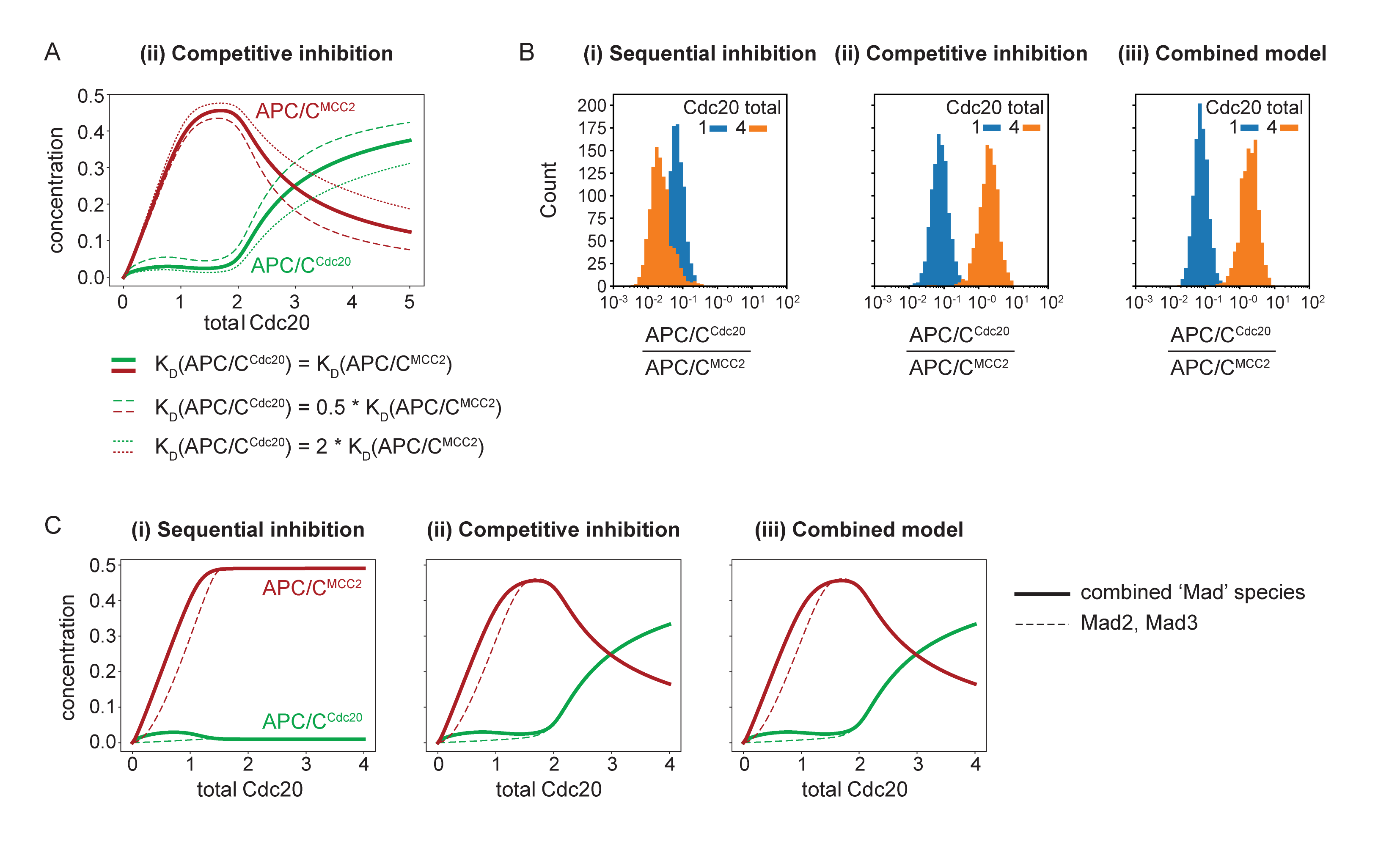

Supplement: S3 Fig — (A) Numerical simulations for the steady state concentrations of APC/CMCC2 and APC/CCdc20 for different total Cdc20 concentrations in the competitive inhibition model (ii), when assuming either the same or different KDs for APC/CCdc20 and APC/CMCC2 formation (reactions 2 and 5, respectively). (B) Robustness of model behavior under parameter variations. The ratio of APC/CCd20 to APC/CMCC2 at Cdc20total = 1 and Cdc20total = 4 was evaluated for 1000 randomly generated parameter sets. In each set, parameters were independently drawn from a log-normal distribution set-value times 10(N(0,0.1)) that roughly varies between 0.5 and 2 times the set-value. (C) Numerical simulations for the steady state concentrations of APC/CMCC2 and APC/CCdc20 for different total Cdc20 concentrations in all three models, either assuming a combined species Mad, which combines both Mad2 and Mad3 (solid lines), or assuming sequential Mad2 and Mad3 binding to Cdc20 (dashed lines). (TIF) [file pcbi.1006449.s005.tif]

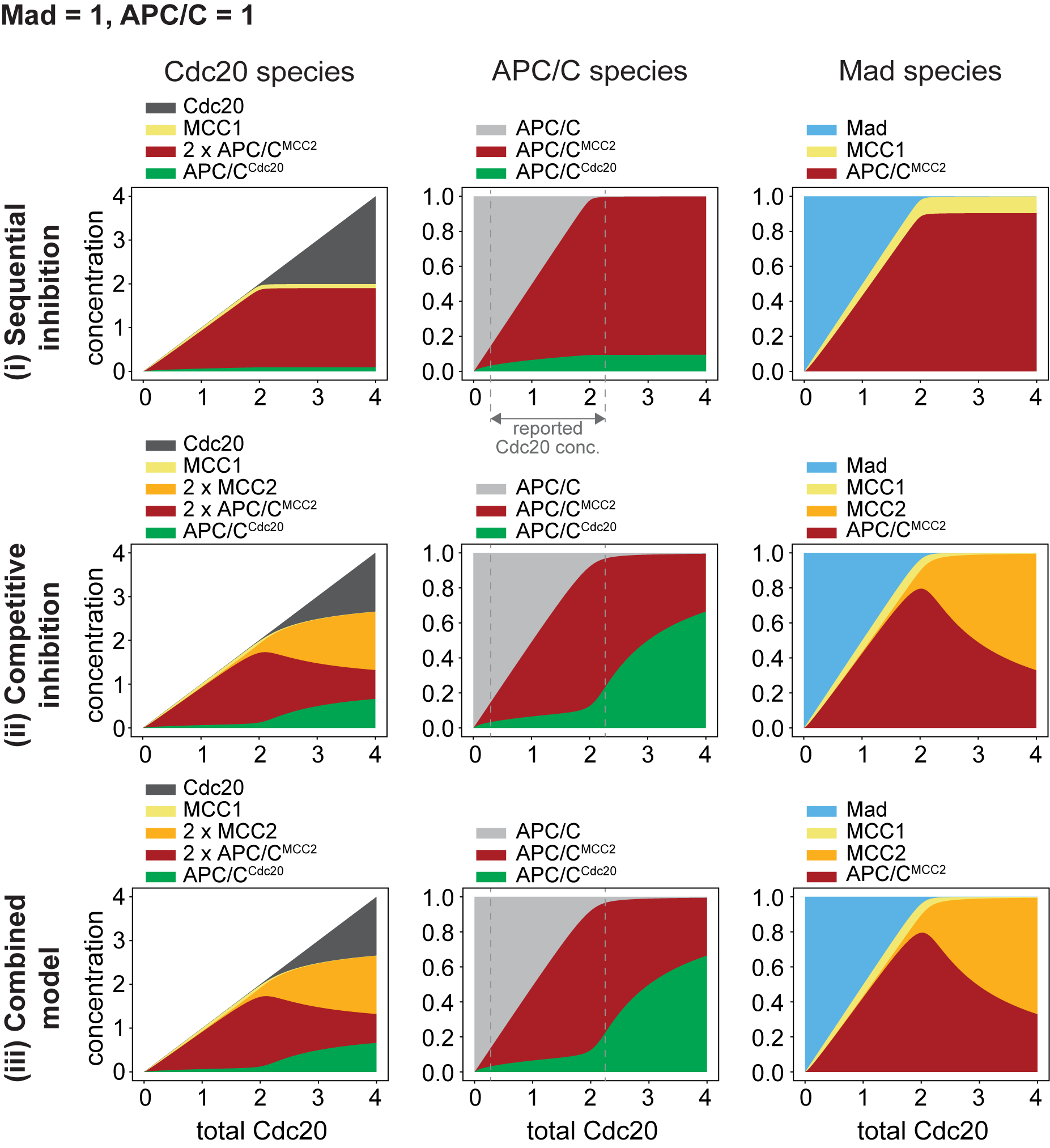

Supplement: S4 Fig — Numerical simulations for the steady state concentration of each species, dependent on the total Cdc20 concentration for each of the networks (i), (ii), and (iii); similar to Fig 4A, except that total Mad and total APC/C concentrations are assumed to be identical (Mad = APC/C = 1). The vertical white dashed lines in the panels for APC/C indicate the physiological Cdc20 range based on reported measurements. (TIF) [file pcbi.1006449.s006.tif]
